# Pathways and Network Based Analysis of Candidate Genes to Reveal Cross-Talk and Specificity in the Sorghum (*Sorghum bicolor* (L.) Moench) Responses to Drought and It's Co-occurring Stresses

**DOI:** 10.3389/fgene.2018.00557

**Published:** 2018-11-20

**Authors:** Adugna Abdi Woldesemayat, Monde Ntwasa

**Affiliations:** Department of Life and Consumer Sciences, College of Agriculture and Environmental Sciences, University of South Africa, Johannesburg, South Africa

**Keywords:** ABA signal transduction pathway, candidate genes, cross-talk, pathways and network analysis, drought response, gene interaction networks, stress combinations, *Sorghum bicolor* L. Moench

## Abstract

Drought alone or in combination with other stresses forms the major crop production constraint worldwide. Sorghum, one of the most important cereal crops is affected by drought alone or in combination with co-occurring stresses; notwithstanding, sorghum has evolved adaptive responses to combined stresses. Furthermore, an impressive number of sorghum genes have been investigated for drought tolerance. However, the molecular mechanism underling drought response remains poorly understood. We employed a systems biology approach to elucidate regulatory and broad functional features of these genes. Their interaction network would provide insight into understanding the molecular mechanisms of drought tolerance and underpinning signal pathways. Functional analysis was undertaken to determine significantly enriched genesets for pathways involved in drought tolerance. Analysis of distinct pathway cross-talk network was performed and drought-specific subnetwork was extracted. Investigation of various data sources such as gene expression, regulatory pathways, sorghumCyc, sorghum protein-protein interaction (PPI) and Gene Ontology (GO) revealed 14 major drought stress related hub genes (DSRhub genes). Significantly enriched genesets have shown association with various biological processes underlying drought-related responses. Key metabolic pathways were significantly enriched in the drought-related genes. Systematic analysis of pathways cross-talk and gene interaction network revealed major cross-talk pathway modules associated with drought tolerance. Further investigation of the major DSRhub genes revealed distinct regulatory genes such as ZEP, NCED, AAO, and MCSU and CYP707A1. These were involved in the regulation of ABA biosynthesis and signal transduction. Other protein families, namely, aldehyde and alcohol dehydrogenases, mitogene activated protein kinases (MAPKs), and Ribulose-1,5-biphosphate carboxylase (RuBisCO) were shown to be involved in the drought-related responses. This shows a diversity of complex functional features in sorghum to respond to various abiotic stresses. Finally, we constructed a drought-specific subnetwork, characterized by unique candidate genes that were associated with DSRhub genes. According to our knowledge, this is the first in sorghum drought investigation that introduces pathway and network-based candidate gene approach for analysis of drought tolerance. We provide novel information about pathways cross-talk and signaling networks used in further systems level analysis for understanding the molecular mechanism behind drought tolerance and can, therefore, be adapted to other model and non-model crops.

## Introduction

Drought is a natural hazard and devastating phenomenon that severely affects agriculture and human life more than any other abiotic stress (Ivits et al., 2014). Sorghum (*Sorghum bicolor* (L.) Monche), a C4 grass and highly genetically variable important major cereal that stands 5th in terms of production is among others increasingly being affected by drought (Abdi et al., 2002; Woldesemayat et al., 2017). The severity of drought on crop production systems has increased and is expected to be exacerbated by the coming years due to increase in the global climate change (Trenberth et al., 2014). While there are a number of other co-varying factors complicate the effect of drought, anthropogenic-induced changes of climate are considered to be the most important. Factors, such as salinity, cold or heat stresses that usually co-exist with drought, play considerable role in compromising plant survival, and productions. As drought becomes sever following the intensity of heat due to increase in the temperature and change in the global climate, and as drought co-occurs with other stresses, investigation of genes, and traits for combined stress tolerance become more important. Under field conditions, individually occurring stresses are rarely observed and crop plants are mostly exposed to a combination of two or more stress types simultaneously. Drought and salinity, drought and extreme temperature, salinity and heat frequently affect many agricultural sectors. Such stress combination occurs in many regions around the globe and causes extreme agricultural losses by many folds than the damage caused by individual stress alone, however these stresses usually are investigated separately (Suzuki et al., 2014). Drought stress with all the co-varying factors not only affect the plant life and productivity, but also contribute to be a more confounded vulnerability risk of global food security.

In the genomic era, advances have been made in analyzing the complex signal transduction process to identify cross-talk between different signaling pathways. Advances in next-generation sequencing technologies have provided novel circumstances for comprehensive medium term and large-scale data analysis for drought-related stress tolerance allowing utilization of deep *de novo* and reference based genome sequences. These efficient and most powerful high-throughput technologies are widely applicable in various studies including discovery of signaling networks and regulatory pathways underlying complex drought-related responses. Molecular analysis of signaling pathways allows identification of proteins that are important in triggering molecular events leading to plant stress response (Yoshida et al., 2014). Plant hormones such as abscisic acid (ABA) play typical role in regulating signaling pathways in response to individual and combined stresses. An integrative role of regulatory hormones could allow modeling signaling mechanisms for specificity and cross-talk between regulatory pathways (Suzuki, 2016). Recent progress has demonstrated that most of these signaling proteins such as MAPK and phytohormones such as ABA were proven to be fundamental targets in metabolic engineering for improving crops' such as sorghum ability to respond to combined stresses (Wani et al., 2016). Previous studies elucidated that plant specific responses to stress combination may require over-expression of unique genesets which are different from the geneset over-expressed in plants exposed to individual stress (Rizhsky et al., 2002). Recently, drought stress response strategies were investigated in sorghum using integrated approaches, whereby genes for tolerance to multiple stresses were identified and evaluated across species (Woldesemayat et al., 2017, 2018b). Although, these investigations, hitherto, have increased our knowledge of stress tolerance mechanism mainly at physiobiochemical level, interaction between multiple individual stresses and stress combinations and the resulting cross-talk between signaling pathways should be studied so as to advance our understanding of molecular mechanism underling stress tolerance associated with natural conditions.

Over the past decades, an increasing number of traits, genes, and proteins that are potentially involved in sorghum drought tolerance have been identified (Abdi and Asfaw, 2005; Dugas et al., 2011; Johnson et al., 2014; Woldesemayat et al., 2018a). However, little or no investigations of molecular mechanisms of signaling and regulatory pathways regarding sorghum tolerance to stress combination was carried out and the few available studies were targeted on stress response individually and rarely in combination to evaluate transcriptional changes (Johnson et al., 2014). In addition, most of the investigations on sorghum stress response cross-talk were focused only on evaluating cross-talk between two individual signaling pathways without including wide range of pathway cross-talk and the protein interactions between them (Shinozaki and Yamaguchi-Shinozaki, 2000; Fujita et al., 2006). Furthermore, recent investigations have evaluated sorghum responses to the effects of drought interaction with other stresses such as effect of high temperature with drought on water relations (Machado and Paulsen, 2001) and mycorrhizal induced effects of combined drought and salt stresses (Cho et al., 2006). All these studies did not provide well organized empirical data that detail the molecular mechanisms underlying signaling and regulatory pathways for tolerance to stress combination, suggesting the need for selection of appropriate strategies to investigate signaling pathways and molecular mechanisms underlying sorghum responses to combined stresses.

To address the concern, designing powerful and biologically plausible approach based on pathway and network analysis is most important to elucidate the molecular mechanisms underlying complex response to combined stresses. While genome-wide expression data analysis allows to discover pathways and networks relevant to drought-related stress phenotypes, establishing comprehensive cross-talk between multiple signaling pathways provides insight for understanding drought stress response mechanism. Such approaches are thought to be instrumental for further insight into complex stress tolerance at the molecular level. It is therefore, important to take a step further to design an approach for understanding the complex mechanism behind signaling pathways in drought-related responses. In the present study, we undertake a comprehensive pathway and network based analysis of candidate genes that were assessed in relation to the PPI network and then compared in terms of response to multiple stresses. The candidate genes and the associated pathways were enriched using functional analysis based on the canonical pathways and Gene Ontology (GO) annotation (Camon et al., 2004). Our approach investigate significantly changed pathways to understand their regulatory functions in drought-related response. We clustered the genes and enriched pathways based on their functions to extract major pathway modules. We further examined the cross-talk quantitatively among significantly enriched pathways to establish a molecular network of stress tolerance and built stress-specific subnetworks using enriched genes specific to drought stress. The genes were further verified using expression data from independent drought experiment. This study provides framework for analyzing candidate gene involved in the complex stress tolerance and signaling networks in other model and non-mode crop plants.

## Materials and methods

### Data recruitment and preprocessing

The candidate genes that were identified to be associated with responses to drought and other stresses such as heat, cold, salt, and oxidative stress were collected from the various data source that included association studies, gene expression profiles from sorghum, from literature search based on studies targeted on plant stress tolerance, and biological regulatory pathways. We identified and prioritized genes for drought tolerance in our previous studies (Woldesemayat et al., 2017, 2018b). We targeted these genes by selecting a list of 133 prioritized genes in the present study and combined with expression data for a comprehensive network and pathway cross-talk analysis using additional and updated data information (Table S1).

### Gene expression profile dataset

The gene expression dataset related to drought stress tolerance, accession numbers GSE30249 (Dugas et al., 2011) and GSE80699 (Fracasso et al., 2016), was retrieved from the Gene Expression Omnibus (GEO) database at the National Center for Biotechnology Information (NCBI). The data contained in GSE30249, on the GPL13779 Illumina Genome Analyzer IIx (Sorghum bicolor) platforms, were composed of 12 drought stressed samples of which 6 were subjected to polyethylene glycol (PEG)-induced osmotic stress and the other 6 to exogenous ABA. Whereas, the data contained in GSE80699 comprised 6 drought stressed samples and the other 6 control samples. In addition, we included candidate genes from stress datasets tested for salt, cold, heat, and oxidative stress tolerance (Woldesemayat et al., 2018b) for comparison, because these stresses usually co-exist with drought stress compounding the effect on agricultural production and plant survival.

### Pathway and protein interaction dataset

The pathway analysis dataset was collected from sorghumCyc (Youens-Clark et al., 2011), sorghum protein-protein interaction (PPI), Kyoto Encyclopedia of Genes and Genomes (KEGG), and GO. The genes collected from each dataset were scored and then ranked using combined scores. This was used to prioritize the genes with the top combined score values for multiple stress tolerance. Enriched genes that were classified into each of the three main GO categories, namely, the Biological Process (BP), Cellular Component (CC), and Molecular Function (MF) were used in the pathway analysis if they were association with drought-related GO terms. The genes that were involved in these GO domains represent group of functionally related geneset with a biological objective to which the interactive proteins contribute and a biochemical activity of interacting proteins linked to the specific sites in the cell. The GO categories based genesets contribute to the pathway cross-talk network as they reflect the biological feature and molecular functional role played by the proteins participating in the interaction and the location of PPI in the cell. We collected sorghum PPI data from the arabidopsis reactome public database (Tsesmetzis et al., 2008) for PPI pairs derived from curated and imported events for model non-arabidopsis plant species based on electronic inference by protein orthology.

### Functional annotation and enrichment analysis

In order to investigate the biological and functional characteristics of genes related to multiple stress tolerance in sorghum, we used a locally installed Blast2GO v. 4.1 (Conesa et al., 2005) and an integrated GO analysis toolkit and database, AgriGO V2 (Du et al., 2010). Blast2GO incorporates locally installed MySQL database and allows evaluation of GO terms enrichment and pathway analysis, where we determined the functional attributes of genes associated with drought-related phenotypes. The GO enrichment was undertaken for the genes grouped under the 3 main GO categories. All the genes included in each canonical pathway were evaluated and the pathways which contain genes overlapping the candidate genes were extracted. Over-representation of GO terms and the probability of genes that overlapped were determined using Fisher's exact test in comparison to the background set based on *p*-values and the False Discovery Rate (FDR). The gene set with lowest *p*-value represent the significance level of enrichment. Terms representing all the GO domains were used in annotation for the enriched ones with adjusted *p*-value (FDR), *p* < 0.05).

### Metabolic pathway analysis

Analysis of biochemical pathways involved in the regulation of genes associated with drought-related responses in sorghum was carried out following the GO enrichment analysis and functional annotation. Enzyme Commission (EC) numbers for the respective enzymes of the key metabolic pathways were assigned to the gene sequences received best blast hits based on the similarity search against non-redundant NCBI protein databases using BlastP search algorithm (Altschul et al., 1990). InterProdomain analysis was also conducted based on a parallel Blast2GO multi-option analysis features. The KEGG orthology (KO) assignments was based on the association of protein sequences encoded by the multi-stress responsive genes with the metabolic pathways. Multi-stress responsive sorghum genes were selected based on the enriched drought-related GO-terms to analyze the metabolic pathways. Significantly enriched pathways and associated major genes which were further categorized into major functional classes were identified using the default parameters of Blast2GO mapping option from the KEGG database (Ogata et al., 1999).

### Analysis of pathway cross-talk network

To investigate the interactions among significantly enriched pathways, we performed pathway cross-talk analysis by describing the overlap between any given pair of pathways using, Jaccard Coefficient (JC) and Overlapping Coefficients (OC), the popularly applied measurements, as indicated below:

$$\text{JC}=\frac{\left|\text{A}\cap \text{B}\right|}{\left|\text{A}\cup \text{B}\right|}\text{ and OC}=\frac{\text{A}\cap \text{B}}{\text{min}(|\text{A}|,|\text{B}|)}$$

where A and B are the number of genes contained in the evaluated pathways. In order to build the pathway cross-talk network, we screened pathways containing multiple genes for cross-talk analysis. We captured the final number of pathways that were filtered by removing the ones containing <3 interacting genes from all the source data-sets, as these may not provide biological information sufficient for proper analysis. We filtered unique gene interactions by removing redundants and self-generated interactions for use in the pathways cross-talk analysis.

Genes overlap between two specific pathways was estimated using Fisher's Exact test (Al-Shahrour et al., 2004). We set the *p*-value based on False Discovery Rate (FDR)-Benjamini-Hochberg (BH) (Benjamini and Hochberg, 1995). *P*-value, FDR < 0.05 was considered as the cut-off threshold value for the pathways to significantly overlap. Following this step, protein interactions between any two pathways were counted by removing the pathway pairs with <3 common genes to make sure sufficient interaction network was established. The pathway pairs were ranked based on the Jaccard and overlap coefficient values. The top scoring pathway cross-talks were selected based on only *p*-value, FDR < 0.01 and were visualized using cytoscape, for complex network analysis (Shannon et al., 2003).

### Analysis of drought-phenotype-specific network

A Cytoscape app/plugin, known as heaviest induced subgraph algorithm (Heinz) (Dittrich et al., 2008) was employed to discover drought-specific subnetwork modules of genes in sorghum PPI networks linked to major drought stress related hub genes (DSRhub genes) involved in the different types of Major Drought Associated Signal Transduction Pathways (MDASTP). This interaction data was expected to include the optimal subnetworks that represent different tissue originated stress related genes. Being grouped into a Steiner-tree problem, a model of Heinz algorithm mainly explores an optimal network and retains subnetworks with maximal score from the PPI network with negative and positive scores. In order to select significant drought specific subnetwork, scores for all the nodes were first estimated and then scores for the edges were defined. The average node degree and local clustering coefficient were also calculated. Based on the edges scores, minimal/maximal spanning tree (MST) was calculated by employing CySpanningTree v.1.1, a Cytoscape app that utilizes Prim's and Kruskal's algorithms to create subnetworks (Shaik et al., 2015). Lastly, the paths between positive and negative nodes were captured and their final scores were determined from which the maximal subnetwork was identified.

## Results

### Functional enrichment network: GO annotation and enrichment analysis

Various drought associated genes that were involved in diverse biological functions were recruited for gene enrichment using GO functional analysis (Table S1). These were grouped into 14 DSRhub genes that potentially contributed to the functional enrichment and interaction network.

**Figure 1:**
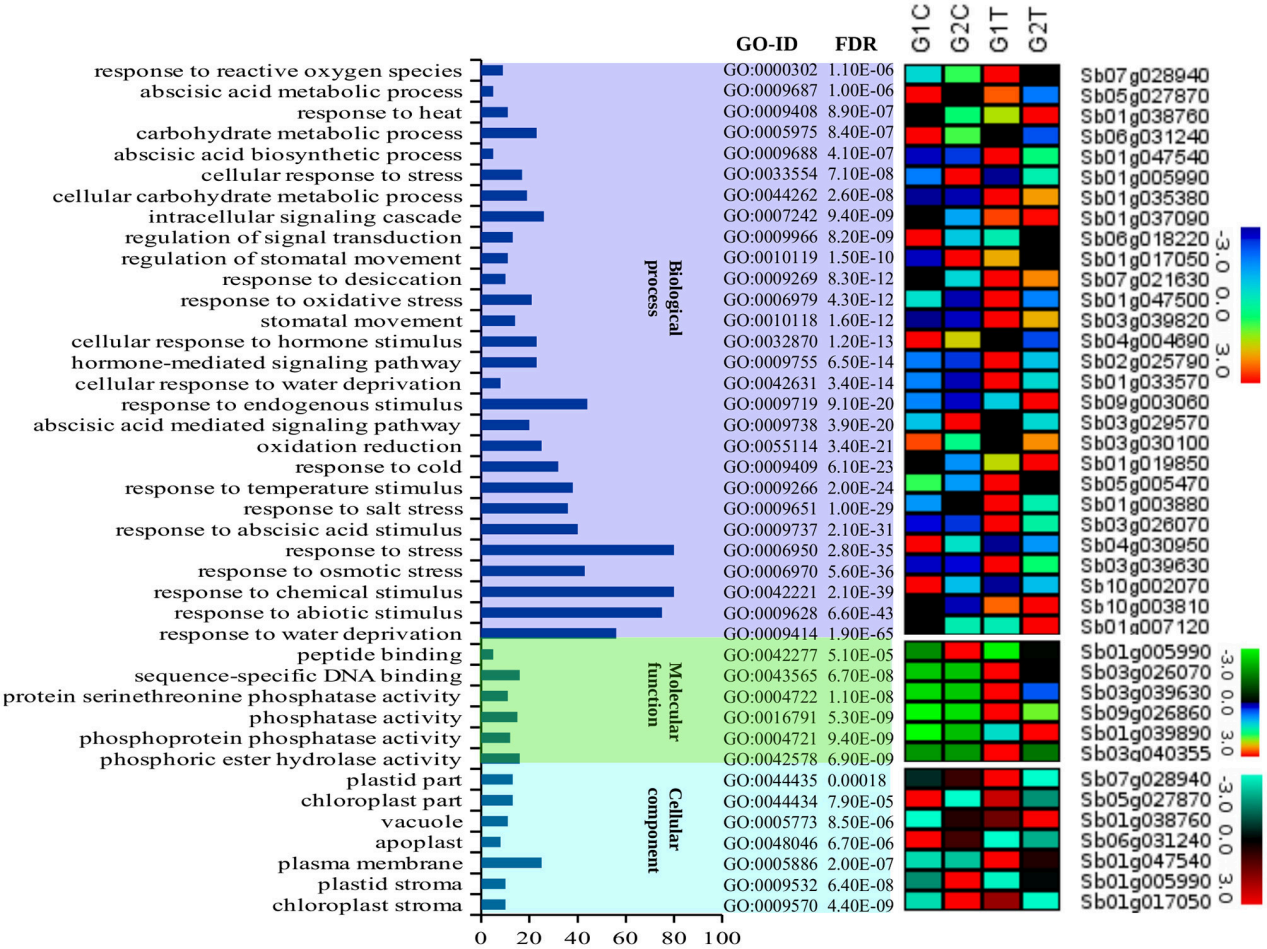
A description of the GO enrichment analysis and corresponding gene expression profile. The figure summarizes a description of the three main GO categories namely biological process, molecular function and cellular component and 41 sub-categories for the GO-terms assigned to the sorghum genes that were involved in drought associated pathways and the corresponding genes that were up and down regulated under drought condition. The different color background shows the different classes of GO categories (x-axis). The number of genes classified in each GO category are shown in the y-axis. To indicate the enrichment level of the GO-terms in the GO categories, we show the associated GO-IDs and the corresponding *p*-values (FDRs). The expression pattern of 41 representative genes to which the corresponding categories of GO-terms were assigned was based on the leaf tissue of 2 sorghum varieties (GSE30249).

AgriGO was employed to assess if the drought stress related genes (DRGs) share specific functional patterns. The GO enrichment analysis revealed a total of 41 significantly enriched sub-categories for the GO-terms assigned to the sorghum DRGs in the 3 main GO categories, namely BP, MF, and CC (Figure 1; Table 1; Table S2). Of all significantly enriched GO terms, 68% were grouped to the biological process including response to water deprivation (GO:0009414; PBH = 1.90E-65); response to desiccation (GO:0009269; PBH = 8.30E-12); response to abiotic stimulus (GO:0009628; PBH = 6.60E-43); response to chemical stimulus (G0:0042221; PBH = 2.10E-39); and response to stress (GO:0006950; PBH = 2.80E-35). It was noted that this category was dominant to which a minimum of 50 DRGs were classified in each sub-category. In addition, GO-terms associated with response to cold and heat and oxidative and salt stresses were significantly enriched (Table S2). In the category of molecular function, 14% enriched GO-terms were accounted to which phosphoric ester hydrolase activity (GO:0042578; PBH = 6.90E-09); phosphoprotein phosphatase activity (GO:0004721; PBH = 9.40E-09); phosphatase activity (GO:0016791; PBH = 5.30E-09); protein serine/threonine phosphatase activity (GO:0004722; PBH = 1.10E-08); sequence-specific DNA binding (GO:0043565; PBH = 6.70E-08) and peptide binding (GO:0042277; PBH = 5.10E-05) were noted to contribute for the classification of a total of 75 DRGs. In the cellular component where 17% GO-terms were grouped, plasma membrane (GO:0005886; PBH = 2.00E-07) was noted to contribute dominantly in the GO category share, where the chloroplast stroma (GO:0009570; PBH = 4.40E-09); plastid stroma (GO:0009532; PBH = 6.40E-08); apoplast (GO:0048046; PBH = 6.70E-06); vacuole (GO:0005773; PBH = 8.50E-06); chloroplast part (GO:0044434; PBH = 7.90E-05); and plastid part (GO:0044435; PBH = 0.00018) collectively contributed to the classification of 90 DRGs. For each enriched GO-term, a gene expression profile was shown for a representative sorghum DRG that was up or down regulated under drought condition, based on the experimental samples from leaf tissues of 2 sorghum varieties, GSE30249 (Figure 1). Based on the GO enrichment analysis, several drought stress response related functional gene categories that were involved in ABA signal transduction, transcription regulation, stress response, hormones signal transduction, and ROS homeostasis were few among others identified (Table S1).

**Table 1:** Description of GO-terms enriched in drought-associated genes.

**GO ID**	**Ontology**	**Description of GO-terms**	**# of genes**	**BG/Ref**	***p*-value**	**FDR**
GO:0006950	P	Response to stress	75	3705	3.90E-044	1.00E-041
GO:0009628	P	Response to abiotic stimulus	60	2423	3.70E-037	4.80E-035
GO:0009414	P	Response to water deprivation	31	374	1.70E-032	1.40E-30
GO:0009415	P	Response to water	31	393	6.90E-032	4.40E-030
GO:0050896	P	Response to stimulus	77	6230	1.40E-030	7.40E-029
GO:0009266	P	Response to temperature stimulus	34	902	5.70E-025	2.40E-023
GO:0042221	P	Response to chemical stimulus	53	3244	2.90E-023	1.10E-021
GO:0009409	P	Response to cold	27	625	3.60E-021	1.20E-019
GO:0009737	P	Response to abscisic acid stimulus	27	664	1.60E-020	4.60E-019
GO:0006970	P	Response to osmotic stress	25	631	9.70E-019	2.50E-017
GO:0010033	P	Response to organic substance	37	2117	2.80E-016	6.40E-015
GO:0009651	P	Response to salt stress	21	536	1.00E-015	2.20E-014
GO:0009725	P	Response to hormone stimulus	32	1601	1.50E-015	2.90E-014
GO:0009719	P	Response to endogenous stimulus	32	1755	1.80E-014	3.30E-013
GO:0070887	P	Cellular response to chemical stimulus	24	941	7.00E-014	1.20E-012
GO:0006979	P	Response to oxidative stress	20	605	1.20E-013	1.90E-012
GO:0009269	P	Response to desiccation	10	71	4.30E-013	6.50E-012
GO:0008152	P	Metabolic process	87	14876	1.30E-012	1.90E-011
GO:0051716	P	Cellular response to stimulus	28	1595	3.00E-012	3.80E-011
GO:0044248	P	Cellular catabolic process	26	1352	3.00E-012	3.80E-011
GO:0003824	F	Catalytic activity	97	13636	3.10E-028	2.70E-026
GO:0016491	F	Oxidoreductase activity	37	2349	7.20E-015	3.20E-013
GO:0016903	F	Oxidoreductase activity	10	145	3.00E-010	8.20E-009
GO:0016620	F	Oxidoreductase activity	9	104	3.70E-010	8.20E-009
GO:0004022	F	Alcohol dehydrogenase (NAD) activity	6	26	1.80E-009	2.20E-008
GO:0016774	F	Phosphotransferase activity	6	25	1.50E-009	2.20E-008
GO:0042624	F	ATPase activity uncoupled	6	26	1.80E-009	2.20E-008
GO:0008553	F	Hydrogen-exporting ATPase activity	6	47	4.30E-008	4.70E-007
GO:0004028	F	3-chloroallyl aldehyde dehydrogenase activity	5	24	6.80E-008	6.60E-007
GO:0016616	F	Oxidoreductase activity	10	271	9.00E-008	8.00E-007
GO:0016614	F	Oxidoreductase activity	10	315	3.50E-007	2.80E-006
GO:0016705	F	oxidoreductase activity	9	252	5.40E-007	3.90E-006
GO:0016765	F	Transferase activity	8	208	1.40E-006	9.40E-006
GO:0016740	F	Transferase activity	39	5115	2.20E-006	1.40E-005
GO:0000287	F	Magnesium ion binding	9	316	3.30E-006	1.80E-005
GO:0015662	F	ATPase activity coupled	6	104	3.40E-006	1.80E-005
GO:0008194	F	UDP-glycosyltransferase activity	9	341	6.00E-006	3.10E-005
GO:0042625	F	ATPase activity coupled	6	141	1.80E-005	8.70E-005
GO:0016757	F	transferase activity	12	768	3.00E-005	0.00013
GO:0016709	F	Oxidoreductase activity	5	93	3.20E-005	0.00013
GO:0005737	C	Cytoplasm	68	9051	1.10E-012	5.00E-011
GO:0044444	C	Cytoplasmic part	54	7660	5.10E-008	1.20E-006
GO:0009507	C	Chloroplast	23	1831	1.90E-007	2.20E-006
GO:0009536	C	Plastid	25	2109	1.50E-007	2.20E-006
GO:0044424	C	Intracellular part	69	12750	5.70E-006	5.40E-005
GO:0005622	C	Intracellular	69	13212	2.60E-005	0.0002
GO:0044464	C	Cell part	80	16988	9.60E-005	0.0005
GO:0044434	C	Chloroplast part	11	729	8.90E-005	0.0005
GO:0005623	C	Cell	80	16988	9.60E-005	0.0005
GO:0005739	C	Mitochondrion	18	1853	0.00015	0.00069
GO:0044435	C	Plastid part	11	793	0.00018	0.00078
GO:0009570	C	Chloroplast stroma	5	142	0.00022	0.00084
GO:0009532	C	Plastid stroma	5	191	0.0008	0.0029
GO:0005829	C	Cytosol	15	1740	0.0019	0.0062
GO:0019866	C	Organelle inner membrane	6	434	0.0058	0.018
GO:0005743	C	Mitochondrial inner membrane	5	325	0.0075	0.022
GO:0031090	C	Organelle membrane	13	1762	0.013	0.037
GO:0031966	C	Mitochondrial membrane	5	381	0.014	0.037
GO:0044429	C	Mitochondrial part	6	546	0.016	0.04
GO:0043231	C	Intracellular membrane-bounded organelle	49	10385	0.019	0.04

### Pathway enrichment analysis and functional classification

**Figure 2:**
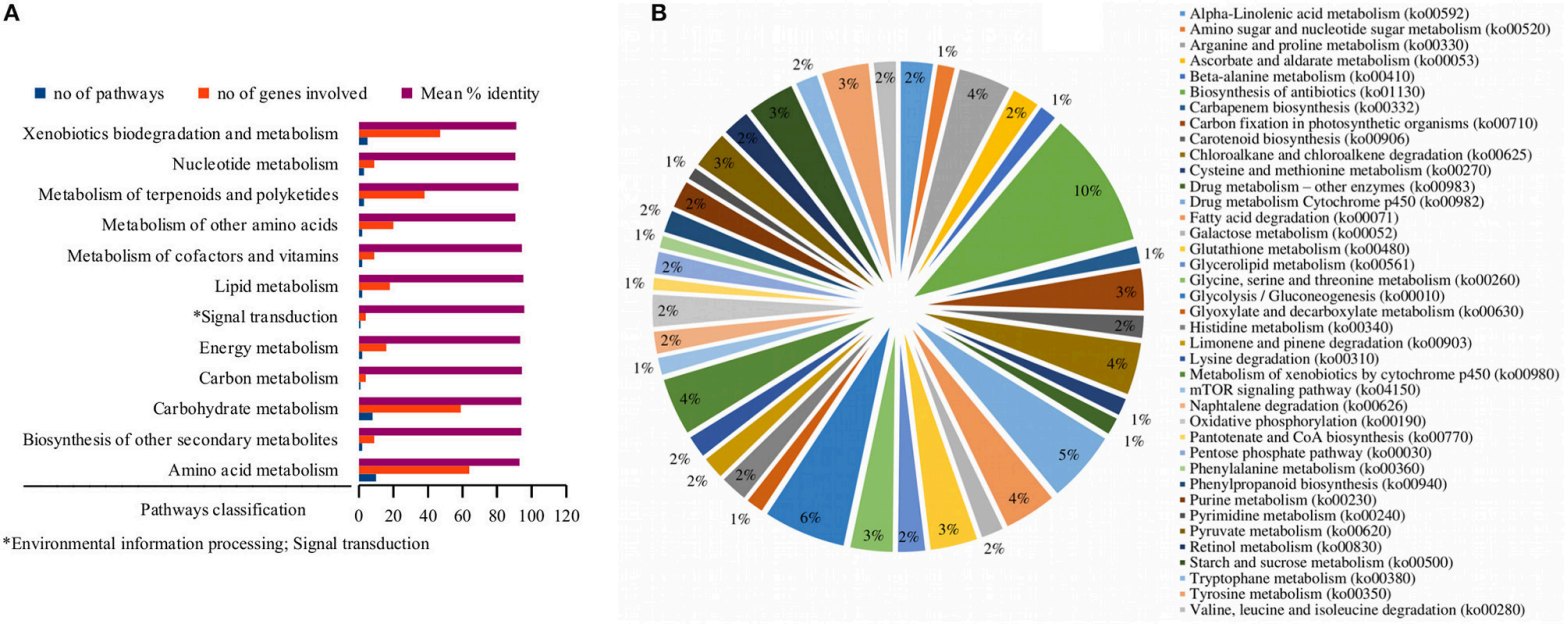
Functional classification of the pathways. While the bar graph in this figure demonstrates the functional classes of the pathways based on specific type of metabolic class to which a particular pathway is grouped **(A)**, the pie chart shows the functional categories of the pathways based on the type and function of drought-related differentially expressed genes that were involved in the pathway **(B)**. The x-axis in **(A)** shows the functional classes of the pathways, while the y-axis shows the number of pathways, the genes involved and mean percent identity of pathways involved in the metabolic classes of the pathways. The distribution of the differentially expressed genes under stress condition among the pathways signifies the functional categories of the pathways in **(B)**. Information on the KEGG pathway annotations and enrichment analysis was based on the Blast2GO using 1e-10 e-value threshold.

A comprehensive investigation of enriched biological pathways for plants exposed to combined effect of stresses may be the key understanding of the mechanism underling cross-talk among stress response pathways. We identified enriched biological pathways for DRGs by performing KEGG pathway analysis using Blast2GO functional annotation platform. The pathways were classified into 13 major functional categories (Figure 2A; Table 2; Table S3), based on the metabolic classes to which they were assigned and based on the associated genes in which they were enriched (Figure 2B). A total of 69 significantly enriched pathways (*p*-value, PBH < 0.01) related to drought responses were identified and annotated with EC numbers to which 77% of the query genes were mapped. Among these, 41 most significantly enriched pathways along their functional classes are shown in Table 2 out of which 33 were selected for pathway cross-talk (Tables S3, S4). It was noted that most of the pathways were related to plant stress signaling transduction involving complex cross-talk between regulatory networks under the combination of stress responses. This suggests that drought response strategies include interaction of multifunctional genes to determine signaling cross-talks. For instance, among others, the metabolic pathways involved in the biosynthesis of drought associate secondary metabolite account for 33.5%, those pathways associated with amino acid metabolism and other primary products represent about 66% (Table S4). While xenobiotics biodegradation and metabolism, metabolism of terpenoids and polyketides, phenylpropanoid biosynthesis (ko00940) and metabolism of xenobiotics by cytochrome p450 (ko00980) represent secondary classes of pathways, metabolic pathways such as α-linolenic acid metabolism (ko00592); carbon fixation in photosynthestic organisms metabolism (ko00710); carotenoid biosynthesis (ko00906); drug metabolism cytochrome p450 (ko00982); glycolysis / gluconeogenesis (ko00010); mTOR signaling pathway (ko04150); and oxidative phosphorylation (ko00190) were few examples among others representing primary pathways involved in the drought response regulation in sorghum (Figure 2; Table S3). Such a categorization of pathways into primary and secondary products may provide metabolic evidence toward stress responses. We identified and selected several metabolic pathways involved in primary and secondary products for further description on the basis of regulatory and target metabolic genes involved in specific and cross-talk between stress responsive pathways.

**Table 2:** Description of key pathways involved in the cross-talk network.

**Pathways**	**Functional class**	**KO path**	**# of genes**	**Mean e-value**	**Mean % identity**
Arganine and proline metabolism	Amino acid metabolism	ko00330	11	0.0	92.0
Ascorbate and aldarate metabolism	Amino acid metabolism	ko00053	6	0.0	93.2
Cysteine and methionine metabolism	Amino acid metabolism	ko00270	4	0.0	93.1
Glycine, serine and threonine metabolism	Amino acid metabolism	ko00260	9	0.0	95.0
Histidine metabolism	Amino acid metabolism	ko00340	6	0.0	98.1
Lysine degradation	Amino acid metabolism	ko00310	5	0.0	92.7
Phenylalanine metabolism	Amino acid metabolism	ko00360	3	0.0	86.0
Tryptophane metabolism	Amino acid metabolism	ko00380	5	0.0	93.3
Tyrosine metabolism	Amino acid metabolism	ko00350	10	0.0	93.1
Valine, leucine and isoleucine degradation	Amino acid metabolism	ko00280	5	0.0	92.7
Carbapenem biosynthesis	Biosynthesis of other secondary metabolites	ko00332	4	0.0	95.0
Phenylpropanoid biosynthesis	Biosynthesis of other secondary metabolites	ko00940	5	0.0	92.8
Amino sugar and nucleotide sugar metabolism	Carbohydrate metabolism	ko00520	4	0.0	91.2
Galactose metabolism	Carbohydrate metabolism	ko00052	5	0.0	91.2
Glycolysis/Gluconeogenesis	Carbohydrate metabolism	ko00010	17	0.0	94.8
Glyoxylate and decarboxylate metabolism	Carbohydrate metabolism	ko00630	4	0.0	95.4
Pentose and glucuronate interconversion	Carbohydrate metabolism	ko00040	6	0.0	93.1
Pentose phosphate pathway	Carbohydrate metabolism	ko00030	5	0.0	96.3
Pyruvate metabolism	Carbohydrate metabolism	ko00620	8	0.0	94.2
Starch and sucrose metabolism	Carbohydrate metabolism	ko00500	10	0.0	95.2
Methane metabolism	Carbon metabolism	ko01200	4	0.0	94.3
Carbon fixation in photosynthetic organisms	Energy metabolism	ko00710	9	0.0	92.9
Oxidative phosphorylation	Energy metabolism	ko00190	7	0.0	93.7
mTOR signaling pathway	Environmental Information Processing; Signal transduction	ko04150	4	0.0	95.8
Alpha-Linolenic acid metabolism	Lipid metabolism	ko00592	7	0.0	96.1
Fatty acid degradation	Lipid metabolism	ko00071	11	0.0	94.5
Pantotenate and CoA biosynthesis	Metabolism of cofactors and vitamins	ko00770	3	0.0	92.9
Retinol metabolism	Metabolism of cofactors and vitamins	ko00830	6	0.0	95.8
Beta-alanine metabolism	Metabolism of other amino acids	ko00410	4	0.0	92.9
Glutathione metabolism	Metabolism of other amino acids	ko00480	10	0.0	87.6
Glycerolipid metabolism	Metabolism of other amino acids	ko00561	6	0.0	91.3
Biosynthesis of antibiotics	Metabolism of terpenoids and polyketides	ko01130	28	0.0	93.5
Carotenoid biosynthesis	Metabolism of terpenoids and polyketides	ko00906	5	0.0	90.9
Limonene and pinene degradation	Metabolism of terpenoids and polyketides	ko00903	5	0.0	92.7
Purine metabolism	Nucleotide metabolism	ko00230	6	0.0	89.4
Pyrimidine metabolism	Nucleotide metabolism	ko00240	3	0.0	92.0
Chloroalkane and chloroalkene degradation	Xenobiotics biodegradation and metabolism	ko00625	11	0.0	94.5
Drug metabolism—other enzymes	Xenobiotics biodegradation and metabolism	ko00983	4	0.0	90.3
Drug metabolism Cytochrome p450	Xenobiotics biodegradation and metabolism	ko00982	15	0.0	89.4
Metabolism of xenobiotics by cytochrome p450	Xenobiotics biodegradation and metabolism	ko00980	12	0.0	96.6
Naphtalene degradation	Xenobiotics biodegradation and metabolism	ko00626	5	0.0	94.7

#### ABA biosynthesis and signaling pathways

**Figure 3:**
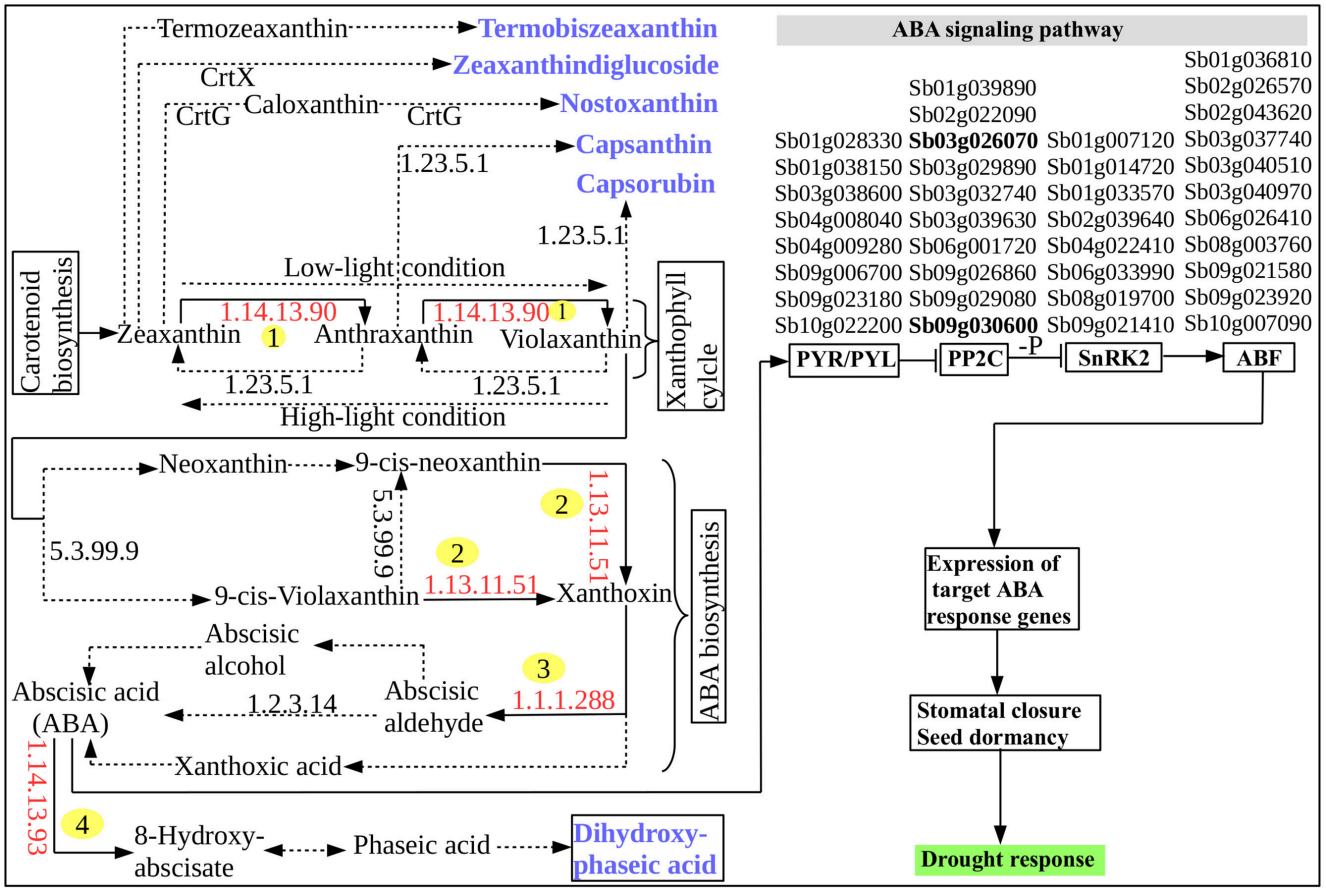
ABA biosynthesis and signaling pathway. The identified enzymes encoded by the *S. bicolor* tissues are indicated by the red enzyme numbers (EC-number) and are labeled with numbers in yellow circle while all other enzymes are indicated by the black EC-numbers and are not labeled. The chemical reaction catalyzed by the enzymes encoded by the *S. bicolor* tissues and that were identified in this study are indicated by black solid arrows. All other reactions and intermediates are indicated with black dashed/dotted arrows. End products are indicated by blue color and bold. A four-column list of ABA or drought-inducible genes that are involved in the ABA signaling occur in the signaling pathway in such a way that each column of the list corresponds from the left to the right to the protein–protein interaction relay channels: PYR/PYL, PP2C, SnRK2, and AREB/ABF for regulating the ion channels and initiating stomatal closure. The 2 highlighted PP2C family genes, “Sb03g026070” and “Sb09g030600,” among others, share, a wide array of functional features with other genes that involve in the cross-talk between ABA biosynthesis and signaling pathway and drought-inducible regulatory network (Table S1).

Based on the carotenoid biosynthesis pathway, interestingly, several DRGs were found to encode key metabolic enzymes that are involved in the biosynthesis and signaling of ABA (Figure 3). The pathway for a biochemical synthesis of a plant specific sesquiterpenoid signaling hormone, ABA, is initiated by a catalytic action of *zeaxanthin* epoxidase (ZEP) [EC:1.14.13.90] (Figure 3) that facilitates the conversion of zeaxanthin and antheraxanthin into violaxanthin (Xiong and Zhu, 2003). With the catalytic action of other enzyme called neoxanthin synthase [EC:5.3.99.9], violaxanthin is converted into 9′-cis-violaxanthin and 9′-cis-neoxanthin. The oxidative cleavage of these into xanthoxin is catalyzed by 9-cis-epoxycarotenoid dioxygenase (NCED) [EC:1.13.11.51] to generate ABA. Xanthoxin dehydrogenase (XDH) [EC:1.1.1.288] catalyzes the conversion of xanthoxin into abscisic aldehyde and contribute to the last step of ABA biosynthesis along with abscisic aldehyde oxidase (AAO) [EC:1.2.3.14] that in turn act on the oxidation of abscisic aldehyde (Seo et al., 2000). Molybdenum cofactor sulfurase (MCSU) involve in this step by catalyzing the production of sulfurylated form of molybdenum cofactor required by AAO (Xiong et al., 2001). However, the accumulation of ABA is regulated by another enzyme, ABA8 hydroxylase [EC:1.14.13.51] because this is important to control the amount of ABA in plants. The sorghum gene CYP707A1 (Sb07g022990) that encodes ABA8 hydroxylase [EC:1.14.13.51] catalyzes this regulatory oxidative and degradative step of ABA. The biochemical pathway of carotenoid biosynthesis provides important metabolic products or metabolites that are used in the ABA biosynthesis. As demonstrated in Figure 3, five caroteinoid products, namely termobiszeaxanthin, zeaxanthindiglucoside, nostoxanthin, capsaxanthin, and capsaruben were produced from the conversion reaction of zeaxanthin, anthraxantin, and violaxanthin in the ABA biosynthesis as well as the dihydroxy-phaseic acid that was produced from the degradative step of ABA. This shows that the integration of the above five major genes, namely, ZEP, NCED, AAO and MCSU and CYP707A1 involve in the regulation of ABA biosynthesis (Figure 3). The role of ABA in regulating plant desiccation tolerance that leads to plants' metabolic and physiological change in response to the drought stress may be associated with the involvement of several drought-activated regulatory and functional genes (Figure 3) that may be induced by ABA itself (Wang et al., 2003). ABA biosynthesis and signaling pathway represents the ABA-mediated responses to drought stress and stomatal regulation.

The molecular mechanism behind ABA signaling pathway involves ABA receptors (PYR/PYL/RCAR), protein phosphatases (PP2C), protein kinases (SnRK2), and the ABA-activated transcription factors and their downstream target genes to initiate stomatal closure (Lee and Luan, 2012) (Figure 3). A total of 37 ABA-inducible genes among others were found to be involved in ABA signal transduction including the 2 known universally expressed genes, Sb03g026070 and Sb09g030600, which were identified under responses to multiple environmental stresses such as cold, drought, heat, and salt stresses (Woldesemayat et al., 2018b). Such widespread functionally diverse genes may be considered critical in sorghum drought tolerance, based on their inhibitory role in the plant hormone signal transduction (Leung and Giraudat, 1998), which is probably associated with their function to encode protein phosphatase 2C (PP2C; EC:3.1.3.16; K14497), an enzyme involved in the negative regulation of the ABA signaling pathway (Nishimura et al., 2010). This finding agrees with a study shown to enhance drought tolerance over multiple stresses using transformed maize ABP9 gene in Arabidopsis (Zhang et al., 2011). All the co-occurring genes involved in the ABA signaling transduction were noted to exist within the same or different protein domains in the relay channel contributing to the regulatory roles in the ABA signaling pathway (Figure 3). Description of DSRhub genes including ABA signaling pathway genes and the corresponding number of pathways enriched and the transcription factor or regulatory genes associated with responses to drought and other stress is provided in Table 3.

**Table 3:** Description of DSRhub genes and the corresponding pathways involved and the transcription factor or regulatory genes categories.

**Description of gene category**	**Acronyms**	**# of pathways**	**# of TF genes**	**# of target genes**
Drought-related supper family of transcription factor	DRTFSF	19	17	19
ABA biosynthesis and Signaling pathways	ABA-BSP	13	9	42
Drought-inducible regulatory protein related genes	DIRPRG	18	13	14
Drought stress related target genes	DSRTG	41	20	43
Heat stress related genes	HSRG	3	6	7
Cold stress related genes	CSRG	11	10	17
Salt stress related genes	SSRG	26	10	13
Oxidative stress related genes	OSRG	5	4	11
Environmental Information Processing and Signal transduction related genes	EIPSTRG	11	8	9
Carbohydrate metabolism related genes	CMRG	34	16	31
Energy and carbon metabolism related genes	ECMRG	25	9	13
Amino acid metabolism related genes	AAMRG	44	13	24
Biosynthesis of secondary metabolites related genes	BSMRG	21	6	9
Other plant hormone related genes	OPHRG	43	16	19

#### Drought stress related inducible regulatory protein related genes (DIRPGs)

This particular hubgene category, contains nine regulatory genes that encode proteins such as late embyogenesis abundant (LEA) 14-A (Sb07g024160); ascorbate peroxidase (APX) (Sb01g038760); basic domain leucine zipper (bZIP) transcription faction TRAB1-like (Sb02g026570); Calcineurin B1 (CaNB1) (Sb01g028750); desertification-related At2g46140-like also called LEA type-2 (Sb02g008820); Ethylen-insensitive 2-like (EIN2) (Sb01g011020); Geranylgeranyl transferase type-I (GGTase 1) superfamily (Sb03g006060); Histidine kinase 1 (HK1) (Sb01g010070). Two additional genes encoding Indol-3-acetaldehyde oxidase (AAO1) (Sb01g005670, Sb02g005200) were highlighted in the inositol phosphate metabolism. Moreover, PP2C related genes such as Sb03g026070; Sb01g039890; Sb02g022090; Sb09g030600 were noted to encode PP2C-6; PP2C-30; PP2C-68, and PP2C-ABI2, respectively. All of these were shown to be associated with drought response regulation (Table S2).

#### Drought-related target genes

Response to desiccation, water deprivation and cellular response to water deprivation related genes accounted for a total of 18 genes that encode for different proteins which are involved in key pathways such as alpha-linolenic and glycerolipid metabolism and pentose and glucuronate interconversion and metabolism were shown to be involved in the sorghum cross-talking response to multiple stresses. Three genes encoding aldehyde dehydrogenase family 3 member a22 and a1 (ALDH3a22) and (ALDH3a14) and family 7 member a1 (ALDH7a1) were highlighted as drought responsive genes such as Sb01g003880, Sb08g004840, and Sb02g025790 (Table S1), respectively, but other desiccation-related genes such as At2g46140-like (LEA type-2) (Sb02g008820) and ethylene-insensitive 2-like (EIN2) (Sb01g011020) were also identified. Three more genes such as Sb02g003090, Sb01g005990, and Sb02g022210 encoding glutathione transferase or s-transferase (GSTs) which were noted to be involved in the metabolism of xenobiotics by cytochrome p450 and glutathione metabolism were found to play active role in multi-stress responses. Chloroplastic Beta-amylase-1 (BAM1), peroxidases 1b and 3 (POX1B, POX3), and Delta-1-pyrroline-5-carboxylate synthase (P5CS1) each being encoded by two genes Sb01g019850 and Sb01g047500, Sb04g004250 and Sb10g027490, and Sb09g022290 and Sb03g039820 were shown to be involved in starch and sucrose metabolism, phenylpropanoid biosynthesis, and carbapenem biosynthesis pathways, respectively (Table S3). Again, 5 genes such as Sb01g031870, Sb01g047540, Sb01g037090, Sb01g048440, and Sb01g031520 each encoding allene oxide cyclase 4 (AOC4), carotenoid cleavage dioxygenase (CCD), galactinol-synthase 2-like (GOLS2), plasma membrane ATPase-like (P-ATPase), and zanthine dehydrogenase (XDH) were shown to play a role in drought responses by involving in the alpha-linolenic acid metabolism, carotenoid biosynthesis, galactose metabolism, oxidative phosphorylation, and purine metabolism, respectively (Table S3).

#### Heat stress related genes

In this category, relatively fewer number of genes were identified that took part in the signaling cross-talk. For instance, 3 genes each encoding heat shock 90 (HSP90) chloroplastic-like (Sb07g028940), Heat-shock 70 (HSP70) (Sb01g017050), and Heat stress transcription factor B2a-like (HsfB2a) (Sb06g025710) and 2 more genes encoding GOLS2 (Sb01g037090 and Sb02g043450) were known to be involved in the galactose metabolism in relation to heat stress response. In addition, 2 more genes such as Sb02g028770 and Sb06g018220 that were shown to encode chtinase (Cas) and ZEP were detected to be involved in the sorghum cellular response to heat. The latter also involve in the carotenoid biosynthesis in response to heat stress.

#### Cold stress related genes

Cold regulated 413 inner membrane 1 (COR413IM1) chloroplastic protein and low-temperature induced protein (LTIP) were shown to be encoded by Sb09g028710 and Sb09g003060, respectively. In response to cold stress, 4 genes such as Sb02g003090, Sb01g005990, Sb02g022210, and Sb02g038130, commonly encoding GSTs, were identified to be involved in the key metabolic pathways, namely drug metabolism cytochrome p450, glutathione metabolism and metabolism of xenobiotics by cytochrome p450. Two more genes (Sb08g005200 and Sb09g002320) each encoding phosphoinositide phospholipase C2-like protein (PLC2) were also known to actively associate with the inositol phosphate metabolism and phosphatidylinositol signaling pathway. Peroxidases 1b and 3, associated with Sb10g027490 and Sb04g004250 was shown to be involved in the phenylpropanoid biosynthesis. Again, GOLS2 being encoded by Sb01g037090 and Sb02g043450 in common was detected to be involved in the galactose metabolism. Furthermore, 3,(2),5-bisphosphate nucleotidase-like protein (BPNT1), in association with 2 genes, namely Sb08g005190, and Sb08g005200, was responsible for sulfur metabolism. It was also noted that 3 additional genes (Sb04g036240, Sb03g030100, and Sb01g031870) that encode probable monogalactosyl diacylglycerols (MGDGs), acid endochitinase-like protein (ECLP) and AOC4 were shown to be involved in the metabolism of glycerolipid, amino sugar, and nucleotide sugar and alpha-linolenic acid metabolic pathways, respectively.

#### Salt stress related genes

Among the set of genes that determine salt stress response in sorghum, two genes, Sb01g038670 and Sb02g030690 encode salt stress induced-hydrophobic peptide (SHP) and salt tolerance protein (STO), respectively. Three genes, Sb02g025790, Sb05g005470, and Sb08g004840 encoding 15 different members of family 3 and family 7 aldehyde dehydrogenase (ALDH3) were detected to represent the major multi-stress responsive component. These genes form a complex biological network by involving in 20 metabolic pathways that are functionally interrelated and associated with drought and salt stress tolerance (Tables S1, S4). Two more genes, Sb09g022290 and Sb03g039820, commonly encoding P5CS1 were known to be involved in the arginine and proline metabolism, carbapenem biosynthesis and biosynthesis of antibiotics for salt stress responses in sorghum. Again 6 genes such as Sb01g037090, Sb04g036240, Sb09g002320, Sb03g030100, Sb02g028770, and Sb01g031870 encoding GOLS2, MGDGs, PLC2, ECLP, Cas, and AOC4, respectively involve in group or separately, in different biochemical pathways such as galactose metabolism, glycerolipid metabolism, inositol phosphate metabolism, phosphatidylinositol signaling system, amino sugar and nucleotide sugar metabolism, and alpha-linolenic acid metabolism.

#### Oxidative stress related genes

It was also interesting to note that 5 genes (Sb01g005990; Sb02g022210; Sb02g038130; Sb02g003090, and Sb10g022780) all of which encoding GSTs actively play a role in the oxidative stress responses through strong association with the metabolism of xenobiotics by cytochrome p450, glutathione metabolism and drug metabolism Cytochrome p450. The genes that encode POX1B/POX3, Sb04g004250, and Sb10g027490, were shown to be involved in the phenylpropanoid biosynthesis. In addition, glycosyltransferases (GTFs) and galactinol-sucrose galactosyltransferase (RFS) being encoded by Sb01g037090 and Sb03g004540, respectively represent active component of antioxidant in sorghum stress tolerance.

#### Environmental information processing and signal transduction related genes

This DSRhub represents nine genes in relation to environmental information processing and signal transduction activities (Table S2). Among these, Sb01g038750 and Sb10g003810 encode mitogen-activated kinase 5 isoform x2 (MAP2K5). The rest seven genes each was shown to encode PLC2 (Sb09g002320); PP2C-9 (Sb09g022410); molybdenum cofactor sulfurase activated kinase 5 isoform x2 (MCSUAK5) (Sb10g026910); phosphopantetheine adenylyltransferase (PPAT) (Sb04g002790); serine threonine-kinase (STK) (Sb01g033570); phosphoglycerate kinase (PGK), cytosolic (Sb04g004690) and phosphoribulokinase (PRK), chloroplastic (Sb04g030950).

#### Carbohydrate metabolism related genes

A total of 26 genes were found to be associated with the biosynthesis of other secondary metabolite activities mainly by involving in carbohydrate metabolism pathway (Table S3). A gene, Sb03g030100, encoding ECLP; 3 genes, Sb01g008730, Sb05g009350, and Sb05g009360, encoding alcohol dehydrogenease 1 (ADH1); 8 genes such as Sb05g005470, Sb08g004840, Sb05g005470, Sb08g004840, Sb05g005470, Sb08g004840, Sb01g003880, Sb02g025790, encoding aldehyde dehydrogenase family 3 members (ALDH3) were noted to actively involve in carbohydrate metabolism. On the other hand, chloroplastic BAM1 being encoded by two genes, Sb01g019850 and Sb01g047500, was also shown to be involved in this specific pathway. Three more genes, Sb03g034680, Sb08g017750 and Sb03g034880, commonly took part in encoding a catalytic subunit, D 5 and 12-like protein called cellulose synthase gene family (CSC) and an additional gene, Sb02g028770, encoding Cas, highlighted drought response mechanism by metabolizing carbohydrates in sorghum. Again, 3 more genes, viz. Sb01g037090, Sb01g037090 and Sb02g043450 that co-act in encoding GOLS2 and another gene (Sb03g004540) that encode RFS were found to be active element in drought response. Four further genes each of which encoding a protein such as glycerate dehydrogenase (GDH) (Sb04g000320), NADP-dependent glyceraldehyde-3-phosphate dehydrogenase (GAPDH) (Sb07g02130); cytosolic phosphoglycerate kinase (PGK) (Sb04g004690), and PLC2 (Sb09g002320) were identified to actively metabolize carbohydrates in response to drought stress in sorghum. RuBisCO and chloroplastic Ribulose phosphate 3 epimerase (RPE/PPE) which were encoded by two genes, Sb05g027870 and Sb01g045920, respectively were associated with drought-related stresses as they involve in carbohydrate metabolism. Again, two more genes, namely Sb01g033060 and Sb10g006330 were detected to co-actively encode sucrose synthase (SUS). A gene, Sb01g035380 that encode UDP-glucose 4 epimerases (UGE) was further identified to be involved in carbohydrate metabolism toward drought stress response in sorghum.

#### Carbon and energy metabolism related genes

Genes biologically attributed to carbon and energy metabolism were found to contribute to drought responses in sorghum. For instance, 13 over expressed genes under drought condition and enriched for drought GO-terms (*p*-value, FDR < 0.05) were shown to be associated with carbon and energy metabolism (Table S3). Among these, 4 genes, namely Sb01g048440, Sb06g031240, Sb07g007610, and Sb10g025470 encoding P-ATPase and two genes, namely Sb08g005190 and Sb08g005200 encoding BPNT1 highlighted drought response in sorghum by activating carbon and energy metabolism. Seven more genes each encoding cytoplasmic aspartate amino transferase (AST) (Sb03g035220), NADH dehydrogenease [ubiquinone] iron-sulfur protein (NDUFS) (Sb02g037780), PGK (Sb04g004690), chloroplastic PRK (Sb04g030950), RuBisCO (Sb05g027870), chloroplastic RPE/PPE (Sb01g045920), RNA polymerase II C-terminal domain (Pol2CTD) (Sb03g040355) were shown to play major role in drought response via maintaining energy balance and carbon metabolism and assimilation (Tables S1, S4).

#### Amino acid metabolism related genes

A total of 41 genes that were associated with amino acid metabolic activities were enriched for drought response (Tables S1, S4). Among these, 20 genes encoding ADH3 were identified to play an integral role in amino acid metabolism associated with drought-related responses. The KEGG pathway analysis highlighted that this set of genes were involved in 17 different metabolic pathways considering both amino acid metabolism and metabolism of other amino acid pathways of which arginine and proline pathways were assigned to 20% of the genes. In addition, 3 genes, namely Sb01g008730, Sb05g009350, and Sb05g009360 encoding ADH1, 6 genes such as Sb02g003090, Sb01g005990, Sb02g022210, Sb02g038130, Sb03g045860, and Sb10g022780 encoding GSTs and 3 other genes (Sb03g039820, Sb09g022290, and Sb09g022290) encoding P5CS1 were shown to take part in amino acid metabolism in response to stresses. Furthermore, 9 genes, namely Sb04g000320, Sb05g014470, Sb10g002070, Sb03g035220, Sb10g026910, Sb07g021630, Sbb0010s007790, Sb03g040355, and Sb04g021990 encoding protein GDH, anosylhomocysteinase (AHCY), arginine decarboxylase-like (ADC), a cytoplasmic protein called AST, MCSUAK5, GAPDH, probable glutathione peroxidase 2 (GPX2), RNA polymerase II C-terminal domain (PolCTD), and chloroplastic 2-Cys peroxiredoxin BAS1 (BAS1), respectively were identified to play prominent role in drought stress responses through maintaining amino acid metabolism (Tables S1, S4).

#### Biosynthesis of secondary metabolites related genes

In this category, a total of 8 genes which were associated with the biosynthesis of other secondary metabolites have shown significant enrichment (*p* < 0.05) for drought terms (Tables S1, S4). Three genes, Sb05g001000, Sb04g004250 and Sb10g027490, encoding POX, 2 more genes (Sb09g022290 and Sb03g039820) encoding P5CS1and four additional genes, Sb01g031520, Sb03g039820, Sb03g035220, and Sb04g021990, encoding xanthine dehydrogenease (XDH), P5CS1, AST and chloroplastic BAS1, respectively were identified to be involved in the biosynthesis of secondary metabolites in response to drought and other stresses in sorghum.

#### Other plant hormone related genes

Genes functionally classified into plant hormones related other than ABA were identified. Five genes (Sb01g008730, Sb05g009350, Sb05g009360, Sb01g031870, and Sb01g003880) which were recognized as group of donors that primarily acting on the CH-OH encode ADH1, AOC4 and ALDH3a22 and mapping to α-linolenic acid metabolism (ko00592), respectively were significantly enriched for drought response. Four more genes, Sb06g018220, Sb01g047540, Sb07g022990, and Sb01g003880, which were mapped to carotenoid biosynthesis pathway encoding ZEP, CCD, NCED, ABA8 hydroxylase (ABA8'OH), and zerumbone synthase (ZSD1) respectively were enriched for drought terms (*p*-value < 0.05). These genes were found to be actively associated with metabolism of terpenoids and polyketides (ko00906). Three other genes (Sb03g035220, Sb05g014470, and Sb03g029570) encoding cytoplasmic AST, AHCY and malate mitochondria-like (MDH) and mapping to cysteine and methionine metabolism were considered drought responsive as they were significantly enriched for drought associated terms. Again, 3 more genes (Sb03g035220, Sb05g005470, and Sb08g004840) encode cytoplasmic AST and ALDH3a15. These genes transferring nitrogenous groups to map to the pathway of phenylalanine metabolism (ko00360) were shown to be enriched for drought response. Furthermore, 5 genes (Sb02g005200, Sb07g021630, Sb02g025790, Sb05g005470, and Sb08g004840) which were mapped to the tryptophane metabolism pathway (ko00380) and acting on the aldehyde or oxo group of donors (Table 4; Table S4) encode the following 5 proteins, namely, AAO1, GAPDH, ALDH7, aldehyde dehydrogenase family 3 member a22 ALDH3a22 and ALDH3a1, respectively. Same genes were detected to be significantly enriched (*P*-value < 0.05) for the drought terms in sorghum.

**Table 4:** Transcription factor involved in drought-related responses.

**Sequence ID**	**Transcription factor description**	**Family of transcription factor**	**E-value**	**% identity**
Sb07g026900.1	TBC1 domain family member 5 homologous	TBC1	0	71.95
Sb01g028870.1	TPA:Myb DNA-binding domain superfamily	Myb	0	74.9
Sb01g039740.1	TPA:RING zinc finger domain superfamily	TPA	7.90E-168	89.85
Sb10g023010.1	Multi-bridging factor 1C (MBF-1C)	MBF	1.00E-093	86.7
Sb03g006450.1	Transcription factor DIVARICATA	DIVARICATA	1.00E-087	78
Sb06g008585.1				
Sb05g021820.1	Myb-related 306	Myb	0	80.5
Sb03g032530.1	Single myb histon 4	Myb	2.70E-167	80.05
Sb05g019540.2	Auxin response factor	ARF	0	88.85
Sb02g026570.1	bZIP transcription faction TRAB1-like	bZIP	0	95.85
Sb03g032290.1	AP1 complex subunit mu-2	AP1	0	98.2
Sb01g011020.1	Ethylen-insensitive 2-like	EI2	0	94.5
Sb04g006970.1	Ethylene-responsive transcription factor	ERTF	0	96.5
Sb02g025080.1	AP2-like ethylene-responsive transcription factor	AP2	0	72.3
Sb04g006970.1	Ethylene-responsive transcription factor	AP2/ERF	1.40E-111	84.4
Sb01g030570.2	MADS-box transcription factor 50 isoform	MADS-box	0	95.2
Sb01g049020.1	MADS Transcription factor, partial	MADS	0	95.85
Sb03g029920.1	Probable WRKY transcription factor 53	WRKY	0	89.75
Sb08g004840.1	Aldehyde dehydrogenase family 3	ALDH	0	95.08
Sb03g006450.1	Transcription factor DIVARICATA	DIVARICATA	1.00E-087	78
Sb01g029220.1	Transcription factorUNE10	UNE10	7.80E-057	87
Sb02g022280.1	WRKY 74 superfamily of TFs having WRKY and zinc finger domains	WRKY	0	82.45

Based on the above analysis, most drought-related metabolic genes that were classified to particular DSRhub were shown to be involved in different stress categories, suggesting functional association of these genes and their internal correlation for increased stress responses, along with their encoded proteins with the sorghum metabolic networks and complex cross-talk between multiple stress responses. This further suggests that the different stress conditions that occur in combination increases the overall association and connectivity of the gene regulation network, whereby such an integrative multisource analysis provides both general understanding of a molecular and regulation network cross-talk and the response specificity to these investigated individual or co-occurring stresses. The presence of uniquely functioning proteins and genes across the board signifies global correlation and gene regulation network connectivity, with demonstrated functional features of specific genes in sorghum drought-related responses. The dynamics of correlation and variance in systems under varying environmental conditions has been already demonstrated (Gorban et al., 2010).

### Cross-talk among significantly enriched pathways

**Figure 4:**
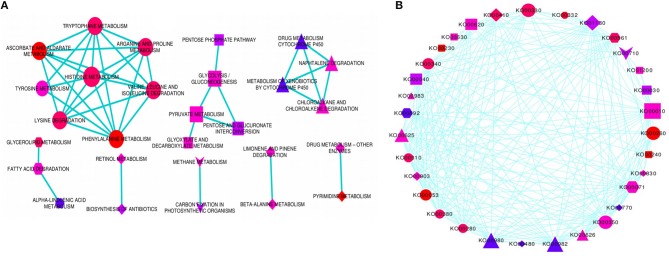
Enriched pathways cross-talk network. The figure shows the enriched pathways cross-talk built based on the gene interaction network for modulated pathways cross-talk network **(A)**, and the circular pathways cross-talk network **(B)**. Three major modules and several minor groups of enriched pathways were linked in cross-talk via DRGs interaction network. The pathways involved in circular cross-talk network were represented by their respective ko identifiers as indicated in **(B)**. Each enriched pathway in the cross-talk contain multiple genes interacting, not <3. Those pathways with <3 genes were disregarded. The significance of any two specific pathways overlap was determined using the *p*-value, FDR < 0.01, as a threshold value, which again limits the size of interacting genes on the cross-talk network. The enriched pathways (*p*-value < 0.01) were selected as an entry for cross-talk network. Each node represents enriched pathway and edges denotes pathway-defined cross-talking relations. The size of nodes corresponds to the number of interacting genes involved in each enriched pathway such that the more the number of interacting genes, the larger is the node. The color of the nodes indicates the significance level (*p*-value) of pathways overlap, the brighter the color (red), the more significant is the pathways overlap. The shape of the nodes corresponds to functional categories (modules) of the pathways, with circle (ellipse) for amino acid metabolism, rectangle for carbohydrate metabolism, triangle for xenobiotics biodegradation and metabolism, hexagon for lipid metabolism, V (inverted triangle) for carbon and energy metabolism, round rectangle for nucleotide metabolism, diamond for metabolism of co-factors and vitamins, and octagon for biosynthesis of other secondary metabolites. Some of the pathways in the modulated pathway cross-talk are not included in the circular pathway cross-talk.

In order to determine the molecular networks and complex interactions of the pathway-defined relationships among regulatory and target DRGs, we analyzed and constructed pathway cross-talk network for 69 significantly enriched pathways. For this purpose, we implemented the following procedure: (1) We determined the number of genes involved in a pathway to be not <3, based on the fact that pathways that are involved in the cross-talk must share sufficient number of DRGs for establishing biologically meaningful molecular network. (2) We maintained the maximum number of genes contained in a pathway as in our particular case, this did not exceed the size of genes that affect the biological information in determining the pathway cross-talk (Li et al., 2008). Based on this, 41 pathways with *p*-value, FDR (PBH) < 0.01 and with not <3 DRGs were selected for the cross-talk. However, it was also found that some of these pathways did not share sufficient number of genes with other pathways. (3) To obtain a substantial biological information, we again considered 3 genes as a limit so as a pathway may share with at least one other pathway, thus, 37 valid pathways remained for further cross-talk analysis. (4) We obtained a total of 222 pathway pairs (edges) which were subjected to ranking order based on the JC and the OC similarity co-efficient scores to select the top 18% edges for constructing the pathway cross-talk. (5) We ended up with 151 pathway pair cross-talk which were grouped into 3 major modules of pathway cross-talk related to their metabolic classes based on their biological function (Figure 4A). The first module is the largest group of pathways related with amino acid metabolism containing 8 members in the cross-talk. These were arganine and proline metabolism, ascorbate and aldarate metabolism, histidine metabolism, lysine degradation, phenylalanine metabolism, tryptophane metabolism, tyrosine metabolism, and valine leucine and isoleucine degradation. While the second module consisting of 5 member pathways, namely, glycolysis/gluconeogenesis, glyoxylate and decarboxylate metabolism, pentose, and glucuronate interconversion, pentose phosphate pathway and pyruvate metabolism is related to the carbohydrate metabolism, the third module consists of only 4 pathways which are related to xenobiotics biodegradation and metabolism; these were chloroalkane and chloroalkene degradation, drug metabolism cytochrome p450, metabolism of xenobiotics by cytochrome p450 and naphtalene degradation. Of note, typical genes such as ALDH, XDH, GPX/GST were noted among others that were involved in the pathway cross-talk (Tables S1, S4). Minor groups of pathways were also detected with strong relations in the pathway cross-talk, some of which were related to carbon and energy metabolism, lipid metabolism and metabolism of terpenoids and polyketides among others (Figure 4; Table S4). Most of the pathways and associated genes linked in cross-talk, were shown to be involved in drought-related responses, in agreement with existing investigation, for instance, amino acid, and cabohydrate metabolism related pathway modules (Zhang et al., 2017) and xenobiotics biodegradation and metabolism related modules (Wang et al., 2016). To our knowledge, the present pathway cross-talk is the first ever constracted in drought stress responses, particularly for cereals, thus provides the basis for understanding the mechanism underlying drought responses. All enriched pathways contained in the major modules and minor groups that linked in cross-talk via interaction network were shown using circular pathways cross-talk network based on ko identifiers (Figure 4B).

### Drought stress response-specific network

**Figure 5:**
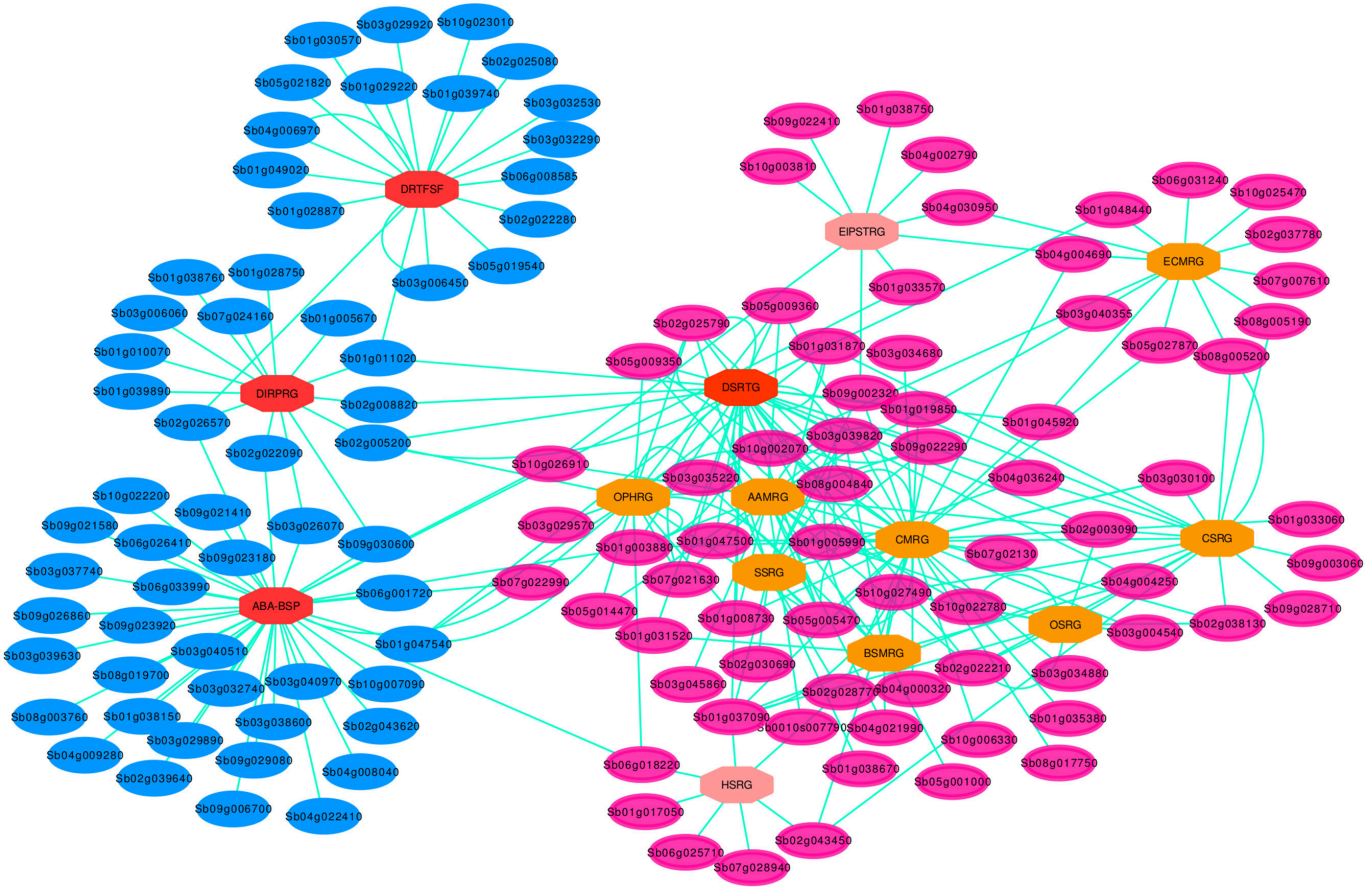
Drought specific PPI sub-network for the key genes involved in the pathway cross-talk network. Blue elliptic nodes represent drought specific pathway genes, MDSGs, while the rose elliptic nodes denote the rest of DRGs, non-MDSGs. The ellipse shape of the nodes shows that all the genes are involved in the drought associated cross-interaction network. Each octagonal node indicate one of the 14 set of pathway DSRhub gene and the color of the octagonal nodes indicate the size of interacting geneset, such that the brighter the color, the greater the number of interacting set of genes. For 14 nodes each represented by DSRhub genes, a total of 266 nodes were denoted by interacting genes that are involved in the pathway defined cross-talking relations.

In order to provide further insight into the DRGs interaction in the pathway cross-talk, we proceeded beyond constructing pathway cross-talk to identify and extract highly modulated drought specific subnetworks from the sorghum PPI network using Heinz algorithm, a Steiner-tree subgroup model. This is because highly modulated subnetworks are target specific and are presumed to contain remarkably likely important individual genes (Razi et al., 2016). Retention of specific subnetworks using Heinz algorithm was mainly based on optimal network with maximal scores from the PPI network. Among 133 DRGs differentially expressed genes that have got full PPI information from the sorghum PPI database (Table S5), 67 were considered in the drought specific subnetwork using Heinz algorithm optimal network based maximal score (Figure 5). The drought specific subnetwork, major drought specific pathway genes (MDSGs) fall into 4 of the 14 main group of pathway genes, DSRhub genes, such as DRTFSF, ABA-BSP, DIRPTGs and DSRTGs. The MDSGs constituted 50.4% of the total DRGs and 28% of the DSRhub genes. In addition, 56 genes were included from the sorghumCycV1.1 (Table S6) of which 45 have shared gene ID in the sorghum PPI network (Table S5) that were mainly associated with drought stress responses. Some of these were shown to be involved in key pathways related to drought stress signaling trunsduction, such as C4 photosynthetic carbon assimilation cycle [EC:4.1.1.31], glycolysis I [4.2.1.11]; gluconeogenesis [4.1.1.31], and sucrose degradation I and III [3.2.1.26] among others (Tables S1, S4). All the DRGs involved in the drought specific subnetwork were tested for their significance using cut off threshold value (*p*-value, FDR < 0.01).

**Figure 6:**
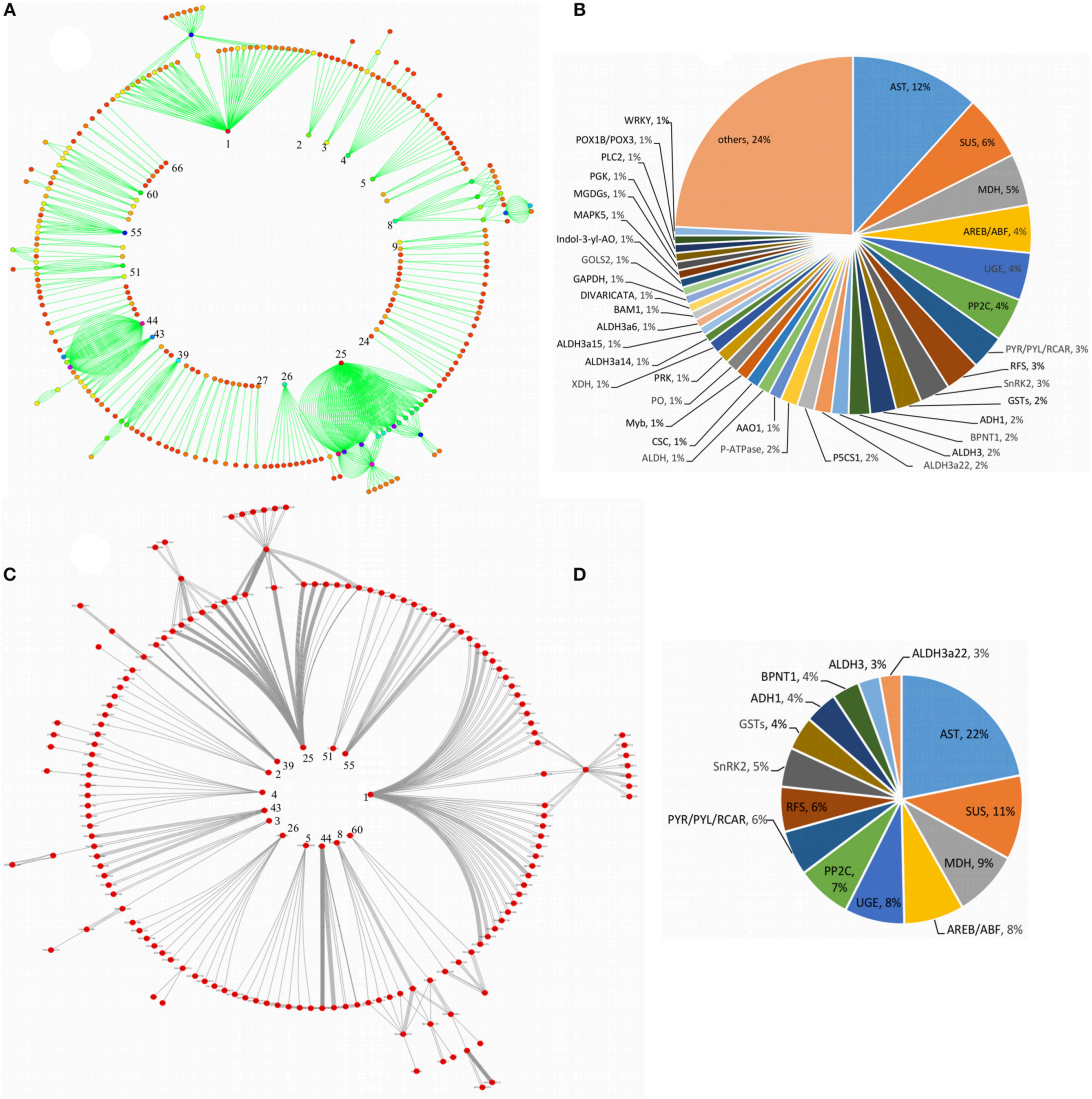
Circular representation of the pathway cross-talk network for key genes involved in the major drought-associated signal transduction and their functional classification. The figure shows a comparative representation of an entire pathways cross-talk network in which inner labeled nodes represented by regulatory genes and the outer labeled nodes represented by target genes **(A)**, the classification of entire target genes into the corresponding number of regulatory genes **(B)**. A radial representation of a sub-pathways cross-talk network specific to drought response is shown by inner nodes represented by regulatory genes, and outer nodes represented by target genes that were extracted from the entire network **(C)** and the corresponding sub-group regulatory genes and the proportion of target genes classified into respective regulatory genes **(D)**. The numbers labeled on the node in-side the circular cross-talk network represent the nodes extracted from the entire network **(A)** to show the drought specific subnetwork **(C)**.

As shown in Figure 6, a comparative circular network representation of a total of 198 DRGs involved in the key pathways were also constructed which was shown by 66 labeled inner nodes represented by regulatory (TF) genes and 710 labeled outer nodes represented by the associated target genes (Figure 6A) and the classification of entire target genes into the corresponding 66 regulatory genes (Figure 6B). Of note, target genes occurring in the same family of regulatory genes but controlled under different classes of regulatory genes were grouped together. Further, a sub-pathways cross-talk network specific to drought response was constructed for 14 nodes represented by regulatory genes and 159 nodes represented by the corresponding target genes (Figure 6C) which were extracted from the entire network (Figure 6A). The corresponding sub-group regulatory genes and the proportion of target genes were classified into each respective regulatory gene family within the sub-group (Figure 6D).

### Expression profiles of DRGs involved in the signaling pathways

**Figure 7:**
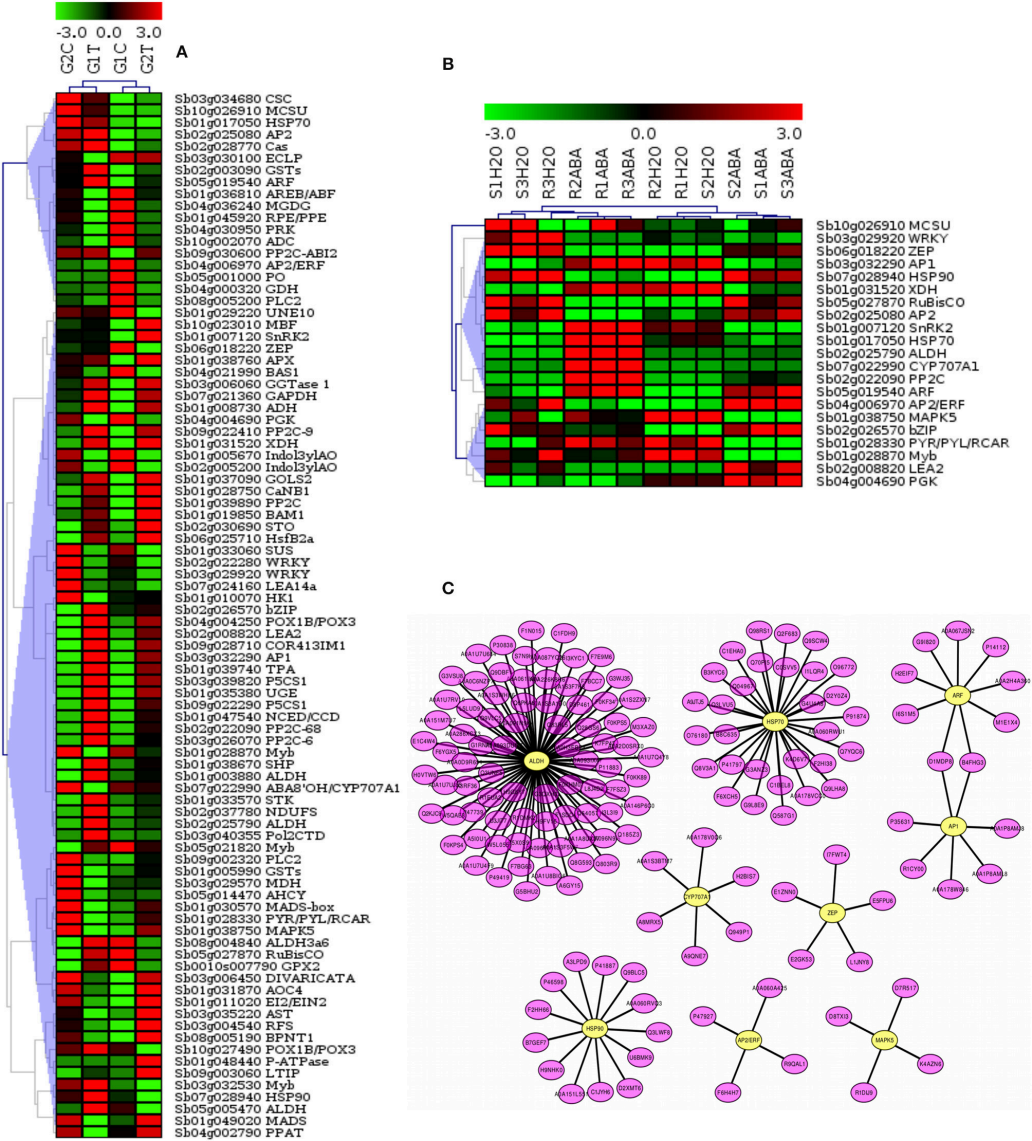
Expression profiles of genes related to major plant stresses categories and the associated transcription factors (TFs) involved in the signaling pathways. The figure shows the hierarchical clustering of target drought expressed genes related to key pathways. Datasets from drought stressed leaf tissues of 2 sorghum varieties, GSE30249 **(A)** and sorghum root and shoot, GSE80699 **(B)** were used to show gene expression profiling. A sub-network of cross-talk based on expression profiles of target and associated regulatory genes related to ABA biosynthesis and other plant related signal transduction pathways is shown in **(C)**. The top regulatory genes involved in the drought-related pathways were indicated in the putative subnetwork for which expression profiles of the associate target genes were shown in **(A)** and **(B)**. Detailed description of the gene expression profiles and related TF genes are provided in Table S7.

To further elucidate the relationship of drought-related gene expression to the signaling pathways in sorghum, we analyzed the gene expression profiles and constructed hierarchical clustering of significant differentially expressed 88 target genes (*p*-value, FDR < 0.01). Drought stressed leaf tissues of 2 sorghum varieties, GSE30249, as indicated in Figure 7A, and 21 selected target genes that were co-expressed in sorghum root and shoot under drought stress, GSE80699 (Figure 7B) were evaluated. A sub-network of regulatory cross-talk is provided based on gene expression profiles and associated transcription factors (TFs) related to ABA biosynthesis and other plant related signal transduction pathways. Top 9 TF genes involved in the drought-related pathways were indicated in the putative regulatory subnetwork (Figure 7C) for which expression profiles of the associate target genes were shown in Figure 7 panel A and B. Detailed description of the gene expression profiles and related TF genes are provided in Table 3; Tables S1, S7. This result shows the relationship between stress induced gene expression and metabolic changes mediated by signaling events in sorghum.

## Discussion

Advancement in global gene expression profiling and high throughput data analysis toward discovering genetic determinants that are potentially involved in the key pathways related to drought stress responses have considerably improved our understanding of the mechanisms underlying drought stress response. In addition, advances in systems biology approaches for integrated functional analysis have contributed to the identification and prioritization of these genetic determinants and the corresponding pathways and networks underlying stress-regulatory cross-talk. In the present study, we introduce a comprehensive pathways and network based analysis of candidate genes in sorghum for functional association with responses to drought-related stresses. In our approach to functional analysis, peculiar biological processes and molecular functions that were associated with specific cellular localizations have been revealed to be linked with DRGs. Importantly, the DRG entries in the GO-enrichment analysis comprehensively accommodated the various sources of DSRhub genes including but not limited to family or supper family of transcription factors, ABA biosynthesis and signaling, drought-inducible regulatory proteins and target genes related to drought, salt, cold, and heat stress responses. This suggests a diverse biological characteristics and functional patterns of sorghum genes pertaining to its natural and improved adaptive responses. Such hub genes were reported previously in response to drought tolerance in various other crops (Hopper et al., 2016; Jaiswal et al., 2018). To this end, the significant association of enriched GO terms such as water deprivation; response to desiccation and salt; response to cold and heat and response to oxidative stresses with most of these genes in sorghum was shown to be empirical evidence for their involvement in regulatory and functional features of drought responses, concordant with the previous results in drought tolerance investigation in sorghum (Dugas et al., 2011; Woldesemayat et al., 2017, 2018b).

Comprehensive analysis of biological pathways in plants under multiple stress situation may be the key step for understanding molecular mechanism underling cross-talk among stress signalings. Pathway investigation revealed significantly enriched cross-talking pathways that were linked to drought and related stress responses. Under drought condition, the function of these signaling pathways and their complex regulatory cross-talk in plants involve intricate process of regulation of gene expression to drive drought tolerance. However, under natural condition where two or more stresses occur concurrently, the process of plant stress response is much more complex involving the interaction of all participating stresses and their signaling pathways, suggesting that plant stress adaptation encompasses wide range of mechanisms including environmental factors and their complex interaction. The classification of these enriched pathways in DRGs into various major metabolic classes suggests their functional diversification in relation to stress tolerance most of which were shown to be related with response to desiccation, salt and oxidative stresses in addition to cold and heat. This further corroborates the presence of close association between such collective stress response phenotypes and the signaling pathways (Zhu, 2002). The identification of primary pathways such as α-linolenic acid metabolism; carbon fixation in photosynthestic organisms metabolism and carotenoid biosynthesis and those secondary pathways such as phenylpropanoid biosynthesis and metabolism of xenobiotics by cytochrome p450 among others signify the close relationship between these biochemical pathways and the drought-related response reactions in sorghum. For instance; the significant involvement of α-linolenic acid metabolism and the metabolism of xenobiotics by cytochrome p450 in plant drought-related stress responses were also noted in other studies (Narusaka et al., 2004; Moradi et al., 2017). The finding of these signaling pathways that involve cross-talking regulatory responses to multiple individuals and combined stresses suggest that the pathways play significant role in modulating wide range of stress tolerance. This is because tolerance across environmental stresses is mainly triggered by complex interacting multi-component signaling pathways to restore cellular function and promote survival (Golldack et al., 2014).

Our analysis, further identified signaling pathways associated with various hub regulatory and target metabolic genes that directly or indirectly involve in the complex interaction network associated with drought-related stress responses. Among these, caroteinoid and ABA biosynthesis and signaling pathways were the central for drought stress related responses of which molecular mechanism behind ABA signaling pathway involves the following four stages of PPI relay channels such as (1) ABA receptors (PYR/PYL/RCAR), (2) protein phosphatases (PP2C), (3) protein kinases (SnRK2), and (4) the ABA-activated transcription factors and their downstream target genes (Umezawa et al., 2009). Of note, in the ABA signaling pathway, a relay of all the protein–protein interaction channels regulates the stomatal closure through controlling the ion channels, depending on the availability of ABA and its efficient binding to its receptors to form ABA-receptor complex (Umezawa et al., 2009, 2010). Among others, ABA-inducible regulatory and target genes were noted to involve in ABA signal transduction pathway which were shown to control ABA signaling by involving in the PP2C inhibited negative regulation of signal transduction pathway. A counter regulation of the signal transduction by protein kinase, SnRK2, provides condition to the last step in the relay. Largely, signal transduction in response to collective stresses of drought and hormone ABA relies on the SnRK2 family of protein kinases in plants (Zhu, 2016). The final response of the ABA signal transduction is the activation of a series of ABA-responsive target genes expression through a regulatory action of the bZIP transcription factor members such as ABA-responsive element binding protein or ABA binding factor (AREB/ABF) (Furihata et al., 2006, 1) to initiate the stomatal closure via depolarization of the guard cells. By and large, these regulatory events enable the guard cells to regulate the movement of water from the cells depending on the level of pressure within the cells allowing the plant to respond to drought stress.

These findings indicate that multiple stress responses that mainly include drought tolerance in sorghum involve complex chain molecular process that include interaction of regulatory proteins and expression of target genes. Importantly, we identified several protein families with distinct enzymatic functions in the metabolic pathways discovered for responses to drought-related stresses in sorghum. Among these, various forms of aldehyde and alcohol dehydrogenases, and NADP-dependent glyceraldehyde-3-phosphate dehydrogenase were few DRGs related to signaling pathways. The presence of these genes and many other protein enzymes such as MAPK, RuBisCO, XDH, NCED, and ZEP that are typically involved in the pathway regulation demonstrate a rich and diverse functional patterns related to complex multi-abiotic stress responses in sorghum.

Based on the pathway cross-talk analysis, 3 major modules of biologically relevant pathway cross-talk and other smaller but strongly linked groups of pathways were identified. Although the size in the modules varies in the number of pathways linked together, it was revealed that the pathways were highly involved in signal transductions related to drought stress responses. The largest module predominantly comprised pathways associated with amino acid metabolism, consistent with the previous observation of stress-induced accumulation of amino acids in plants (Krasensky and Jonak, 2012; Zhang et al., 2017). Among others, a pathway involved in proline metabolism is a remarkable evidence for biological process underlying plant stress tolerance. Proline accumulation and a resulting increased tolerance in many plant species under stress conditions such as drought and high salinity has been well documented (Yamada et al., 2005), because proline primarily acts as an osmolyte and a molecular chaperone to protect cells from damage or stress induced apoptosis (Szabados and Savouré, 2010). Conversely, impaired induction of proline accumulation by gene knock-out was shown to render plants more sensitive to stress (Székely et al., 2008), suggesting a paramount role of the genes regulating amino acid level in plants. While the second module that was associated with carbohydrate metabolism consists of pathways such as glycolysis /gluconeogenesis, glyoxylate and decarboxylate metabolism, pentose and glucuronate interconversion, pentose phosphate pathway, and pyruvate metabolism, the last module being associated with xenobiotics biodegradation and metabolism typically includes chloroalkane and chloroalkene degradation, drug metabolism Cytochrome p450, metabolism of xenobiotics by cytochrome p450 and naphtalene degradation all of which demonstrated strong association with major genes participated in the network interaction. As such, an association between drought tolerance and accumulation of solutes such as carbohydrates, amino acids and lipids toward contributing to osmotic adjustments has been documented (Chen and Jiang, 2010), revealing most of the identified pathways contributed to widely known biochemical mechanisms of drought tolerance. Besides, the involvement of peculiar genes such as GPX/GST in the drug metabolism cytochrome p450 and metabolism of xenobiotics by cytochrome p450 pathways provides inference to the association of drought tolerance with xenobiotics through increased role in detoxification and biodegradative systems, in support of the previous report (Wang et al., 2016). Eventually, it was noteworthy to gather all the genes together that were closely connected with and imparted to the cross-talk. The most abundantly occurring genes across the board included ALDH, HSP70, HSP90, plant specific genes such as auxin response factors (ARF), APETALA1 (AP1), APETALA2/ethylene response factor (AP2/ERF) TF gene, ZEP, and MAPK5 among others, suggesting these genes might be more potential targets in the enhancement of drought tolerance. Of note, the pathway pair of carbon and energy metabolism, lipid metabolism and metabolism of terpenoids and polyketides among others represent useful member of the pathway cross-talk in mediating drought response signaling, although these were not included in the three major modules. Of course, based on the pathway pairs selection criteria that only included the top performers, some pathway pairs that might be of importance were missed from the cross-talk. However, the most perturbed pathways in drought-related stresses mainly remain to be arganine and proline metabolism, glycolysis /gluconeogenesis and drug metabolism Cytochrome p450, metabolism of xenobiotics by cytochrome p450 and naphtalene degradation where the genes associated with these pathways were prominent component of interaction network for the biological processes associated with drought tolerance. A drought-specific subnetwork that was extracted from the pathway pairs cross-talk network revealed major genes involved in the key signaling pathways and interaction network associated with response to drought stress, in favor of biological evidence that more often support for pathway cross-talk occurs between some subsets rather than the whole sets of pathway genes (Wang et al., 2013). Among these genes, those additionally recruited from sorghumCyc that were also included in the sorghum PPI network were shown to be involved with drought stress responses. For example, transcriptionally activated by the MYB transcription factors gene family, a multifaceted gateway enzyme, phenylalanine ammonia-lyase (PAL), can involve in the regulation of the biosynthesis of multiple biochemical compounds such as phenylpropanoids, suberin, and salicylic acid among others (Zhang and Liu, 2015; Mondal and Roy, 2018), suggesting the importance of this major gene in the development of drought tolerance. Phosphoenolpyruvate carboxykinase (PEPCK), a rate-limiting enzyme in gluconeogenesis and malate metabolism can involve in drought response by modulating the effect of photosynthesis through regulation of stomatal movement (Penfield et al., 2012).

It was interesting to note the different types of differentially expressed genes for which significantly enriched pathways and cross-talk network were identified that contributed to the functional insight of biological process associated to drought tolerance. As such, large-scale gene expression data analysis, based on multiple gene set-approach is suggested to understand pathway cross-talk (Wang et al., 2013). An assessment for global interrelationship of the constructed networks for drought and other stress related genes as compared to the reference or control set revealed that the 133 drought expressed and functionally enriched geneset demonstrated an internal correlation within the entire set, thus, representing a specific entity for extraction that were selectively enriched for the commonly occurring co-regulated modules, in agreement with the previous finding for a gene regulation networks (Censi et al., 2011). In this study, several sets of co-expressed and co-regulated genes were shown to be linked with drought associated transcription factor genes related to ABA biosynthesis and other plant related signaling pathways, suggesting the close relationship between stress induced gene expression and metabolic changes mediated by signaling events in sorghum.

## Conclusions

Factors that govern the physio-biochemical and molecular processes underpinning drought tolerance are complex. To elucidate this, we designed a systems biology approach for integrated and comprehensive analysis of molecular pathways and candidate genes related to drought tolerance. The results show distinct drought response-related signal pathways and biological process that play key roles in the molecular mechanism of drought responses that stemmed from integrated analysis of functional GO, pathway enrichment, pathway cross-talk, gene expression profiling, and gene/protein interaction network. This analysis revealed that ABA and other plant hormone signaling pathways might play key roles in the improvement of sorghum stress tolerance, considering that our prioritized empirically identified top candidate genes were typical of drought response displaying an increased global correlation between them. The major functional modules in the pathway cross-talk and other substantially linked groups of interaction networks were shown to be significantly associated with drought tolerance. Remarkably, the extraction of drought response-specific subnetwork revealed that both the initial DSRhub genes and additional candidates were shown to be involved in key pathway signals related to drought tolerance. This finding represents original information and could be used as a reference in future systems level comprehensive analysis, thus provides insight into understanding the mechanisms underlying responses to combined stresses. Further, investigation of cross-talk among pathways in this study could potentially shed new light on understanding the physiology of drought response in model and non-model cereal crops.

## Availability of data and materials

All data generated and analyzed during this study are included in this article. All other datasets used in the study are publicly available and can be accessed following the instruction in the “data acquisition and preprocessing” section.

## Author contributions

AW conceived of the study, designed and performed the analysis, interpreted the data, wrote, and revised the manuscript. MN contributed to editing and the final preparation of the manuscript.

### Conflict of interest statement

The authors declare that the research was conducted in the absence of any commercial or financial relationships that could be construed as a potential conflict of interest.

## Supplementary material

Table S1 List of major drought and related stress response regulatory or functional genes (DSRhub genes) used in the pathway cross-talk analysis.

Table S2 List of GO-accessions and associated GO-terms enriched for drought and related stresses response genes.

Table S3 In put genes for circular enriched pathways-cross-talk.

Table S4 Detailed description of metabolic pathway analysis.

Table S5 Detailed description of sorghum PPI data used as an input for pathway network analysis.

Table S6 Input data for initial and additional genes used in constructing drought specific subnetwork based on sorghumCyc that contains gene identifiers in the sorghum PPI database.

Table S7 Significantly expressed genes that were involved in the pathway cross-talk analysis.

## Abbreviations

ABA: Abscisic Acid
ABRE: ABA-dependent cis-acting element or ABA-Responsive Element Repeat
AREBs: ABA-responsive element-binding proteins
DRGs: Drought stress related genes
DSRhub genes: One of the 14 major drought and related stress response genesets
FDR: False Discovery Rate
GEO: Gene Expression Omnibus
GWAS: Genome Wide Association Studies
MDSGs: Major drought specific genes
NCBI: National Centre for Biotechnology Information
MAPK: Mitogen-activated protein kinases
MCSU: Molybdenum cofactor sulphurase
NCED: 9 cis epoxycarotenoid dioxygenase
ZEP: Zeaxanthin oxidase.
