# Supplementary material for: Pathways and Network Based Analysis of Candidate Genes to Reveal Cross-Talk and Specificity in the Sorghum (Sorghum bicolor (L.) Moench) Responses to Drought and It's Co-occurring Stresses
Source: Front Genet. 2018 Nov 20;9:557. doi: 10.3389/fgene.2018.00557 (PMC6255970; doi:10.3389/fgene.2018.00557)
Supplement: Table S3 — In put genes for circular enriched pathways-cross-talk. [file Table_3.PDF]

.

**Additional file 3: In put genes for circular enriched pathways-crosstalk**

| | | | | | | | | | | | | | | | | | | | | |
|---|---|---|---|---|---|---|---|---|---|---|---|---|---|---|---|---|---|---|---|---|
| ko00592 | Sb01g008730.3 | Sb01g008730.2 | Sb01g008730.1 | Sb05g009350.1 | Sb05g009360.1 | Sb01g031870.1 | Sb01g003880.1 | | | | | | | | | | | | | |
| ko00330 | Sb09g022290.1 | Sb03g035220.2 | Sb09g022290.2 | Sb07g021630.1 | Sb03g039820.2 | Sb03g039820.1 | Sb10g002070.1 | Sb02g025790.1 | Sb05g005470.1 | Sb08g004840.1 | Sb02g005200.1 | | | | | | | | | |
| ko00053 | Sb07g021630.1 | Sb02g025790.1 | Sb05g005470.1 | Sb08g004840.1 | Sb02g005200.1 | Sb01g038760.1 | | | | | | | | | | | | | | |
| ko00410 | Sb07g021630.1 | Sb02g025790.1 | Sb05g005470.1 | Sb08g004840.1 | | | | | | | | | | | | | | | | |
| ko01130 | Sb01g045920.2 | Sb03g035220.2 | Sb07g021630.1 | Sb04g004690.2 | Sb03g039820.2 | Sb04g004690.1 | Sb05g027870.1 | Sb01g003880.1 | Sb01g008730.3 | Sb01g008730.2 | Sb01g008730.1 | Sb03g040355.1 | Sb05g009350.1 | Sb05g009360.1 | Sb02g029780.1 | Sb02g025790.1 | Sb05g005470.1 | Sb03g034000.1 | Sb08g004840.1 | Sb09g022290.1 |
| ko00332 | Sb09g022290.2 | Sb03g039820.2 | Sb03g039820.1 | Sb09g022290.1 | | | | | | | | | | | | | | | | |
| ko00710 | Sb01g045920.2 | Sb03g035220.2 | Sb04g030950.1 | Sb04g004690.2 | Sb04g004690.1 | Sb04g030950.2 | Sb05g027870.1 | Sb03g029570.1 | Sb03g008050.1 | | | | | | | | | | | |
| ko00625 | Sb07g021630.1 | Sb01g003880.1 | Sb01g008730.3 | Sb01g008730.2 | Sb01g008730.1 | Sb05g009350.1 | Sb05g009360.1 | Sb02g025790.1 | Sb05g005470.1 | Sb08g004840.1 | Sb02g005200.1 | | | | | | | | | |
| ko00983 | Sb01g031520.1 | Sb01g031520.2 | Sb04g030950.1 | Sb04g030950.2 | | | | | | | | | | | | | | | | |
| ko00982 | Sb02g004310.1 | Sb06g001960.1 | Sb10g022780.1 | Sb03g045860.1 | Sb01g008730.3 | Sb01g008730.2 | Sb01g008730.1 | Sb05g009350.1 | Sb05g009360.1 | Sb01g005990.1 | Sb02g003090.1 | Sb02g022210.1 | Sb05g005470.1 | Sb08g004840.1 | Sb01g003880.1 | | | | | |
| ko00071 | Sb07g021630.1 | Sb01g003880.1 | Sb01g008730.3 | Sb01g008730.2 | Sb01g008730.1 | Sb05g009350.1 | Sb05g009360.1 | Sb02g025790.1 | Sb05g005470.1 | Sb08g004840.1 | Sb02g005200.1 | | | | | | | | | |
| ko00480 | Sb04g021990.2 | Sb10g022780.1 | Sb03g045860.1 | Sb04g021990.1 | Sb0010s007790 | Sb01g005990.1 | Sb02g003090.1 | Sb02g022210.1 | Sb02g038130.1 | Sb01g038760.1 | | | | | | | | | | |
| ko00561 | Sb07g021630.1 | Sb02g025790.1 | Sb04g036240.1 | Sb05g005470.1 | Sb08g004840.1 | Sb02g005200.1 | | | | | | | | | | | | | | |
| ko00260 | Sb04g000320.2 | Sb04g000320.3 | Sb01g003880.1 | Sb01g008730.3 | Sb01g008730.2 | Sb01g008730.1 | Sb03g040355.1 | Sb05g009350.1 | Sb05g009360.1 | | | | | | | | | | | |
| ko00010 | Sb07g021630.1 | Sb04g004690.2 | Sb04g004690.1 | Sb01g008730.3 | Sb01g008730.2 | Sb01g008730.1 | Sb05g009350.1 | Sb05g009360.1 | Sb02g025790.1 | Sb05g005470.1 | Sb08g004840.1 | Sb02g005200.1 | Sb05g004590.1 | Sb05g004590.1 | Sb03g008050.1 | Sb01g010280.1 | Sb01g003880.1 | | | |
| ko00630 | Sb04g000320.2 | Sb04g000320.3 | Sb05g027870.1 | Sb03g029570.1 | | | | | | | | | | | | | | | | |
| ko00340 | Sb02g005200.1 | Sb03g035220.2 | Sb07g021630.1 | Sb02g025790.1 | Sb05g005470.1 | Sb08g004840.1 | | | | | | | | | | | | | | |
| ko00903 | Sb07g021630.1 | Sb02g025790.1 | Sb05g005470.1 | Sb08g004840.1 | Sb02g005200.1 | | | | | | | | | | | | | | | |
| ko00310 | Sb07g021630.1 | Sb02g025790.1 | Sb05g005470.1 | Sb08g004840.1 | Sb02g005200.1 | | | | | | | | | | | | | | | |
| ko00980 | Sb10g022780.1 | Sb03g045860.1 | Sb01g003880.1 | Sb01g008730.3 | Sb01g008730.2 | Sb01g008730.1 | Sb05g009350.1 | Sb05g009360.1 | Sb01g005990.1 | Sb02g003090.1 | Sb02g022210.1 | Sb05g005470.1 | Sb08g004840.1 | | | | | | | |
| ko01200 | Sb03g040355.1 | Sb03g029570.1 | Sb05g004590.1 | Sb03g008050.1 | | | | | | | | | | | | | | | | |
| ko00626 | Sb01g003880.1 | Sb01g008730.3 | Sb01g008730.2 | Sb01g008730.1 | Sb05g009350.1 | Sb05g009360.1 | | | | | | | | | | | | | | |
| ko00770 | Sb02g004440.1 | Sb01g008730.1 | Sb04g002790.1 | | | | | | | | | | | | | | | | | |
| ko00040 | Sb01g045920.2 | Sb07g021630.1 | Sb02g025790.1 | Sb05g005470.1 | Sb08g004840.1 | Sb02g005200.1 | | | | | | | | | | | | | | |
| ko00030 | Sb01g045920.2 | Sb07g021630.1 | Sb05g004590.1 | Sb03g008050.1 | Sb01g010280.1 | | | | | | | | | | | | | | | |
| ko00360 | Sb03g035220.2 | Sb05g005470.1 | Sb08g004840.1 | | | | | | | | | | | | | | | | | |
| ko00230 | Sb01g031520.1 | Sb04g024570.1 | Sb01g031520.2 | Sb01g005670.1 | Sb08g017600.1 | Sb01g010280.1 | | | | | | | | | | | | | | |
| ko00240 | Sb04g030950.1 | Sb04g030950.2 | Sb08g017600.1 | | | | | | | | | | | | | | | | | |
| ko00620 | Sb04g000320.2 | Sb04g000320.3 | Sb07g021630.1 | Sb02g025790.1 | Sb05g005470.1 | Sb08g004840.1 | Sb02g005200.1 | Sb03g029570.1 | | | | | | | | | | | | |
| ko00830 | Sb01g003880.1 | Sb01g008730.3 | Sb01g008730.2 | Sb01g008730.1 | Sb05g009350.1 | Sb05g009360.1 | | | | | | | | | | | | | | |
| ko00380 | Sb02g005200.1 | Sb07g021630.1 | Sb02g025790.1 | Sb05g005470.1 | Sb08g004840.1 | | | | | | | | | | | | | | | |
| ko00350 | Sb01g003880.1 | Sb03g035220.2 | Sb07g021630.1 | Sb01g008730.3 | Sb01g008730.2 | Sb01g008730.1 | Sb05g009350.1 | Sb05g009360.1 | Sb05g005470.1 | Sb08g004840.1 | | | | | | | | | | |
| ko00280 | Sb07g021630.1 | Sb02g025790.1 | Sb05g005470.1 | Sb08g004840.1 | Sb02g005200.1 | | | | | | | | | | | | | | | |
